# Multifunctional intercalants create stable subnanochannels in MoS_2_ membranes for wastewater treatment

**DOI:** 10.1038/s41467-025-58409-x

**Published:** 2025-09-24

**Authors:** Hao Zhang, Ming Yong, Ting Hu, Yuan Kang, Zhuyuan Wang, Zhonghao Xu, Xuefeng Li, Xin Sun, Lijun Guo, Fangmeng Sheng, Xiangkang Zeng, Zhikao Li, Xingya Li, Huanting Wang, Tongwen Xu, Xiwang Zhang

**Affiliations:** 1 UQ Dow Centre for Sustainable Engineering Innovation, School of Chemical Engineering, The University of Queensland, St Lucia, QLD Australia; 2 Key Laboratory of Precision and Intelligent Chemistry, School of Chemistry and Materials Science, University of Science and Technology of China, Hefei, PR China; 3 Department of Chemical & Biological Engineering, Monash University, Clayton, VIC Australia; 4 National Engineering Research Center of Clean Technology in Leather Industry, Sichuan University, Chengdu, PR China; 5 ARC Centre of Excellence for Green Electrochemical Transformation of Carbon Dioxide (GETCO2), The University of Queensland, St Lucia, QLD Australia

**Keywords:** Two-dimensional materials, Chemical engineering

## Abstract

MoS_2_ nanosheets, featuring high chemical and mechanical stability, offer immense promise as building blocks for high-performance two-dimensional (2D) membranes. However, engineering these membranes to achieve tailored channel dimensions and chemistry while maintaining sufficient stability remains a significant challenge, impeding their real-world applications. Herein, we demonstrate the multifunctionality of polymeric quaternary ammoniums as intercalants in MoS_2_ membranes, enabling the creation of selective, stable 2D subnanochannels in MoS_2_ membranes. These intercalants fulfil three key roles: they define and secure the channel width at ~5 Å without disrupting the channel order, impart substantial positive charges to regulate the microenvironment within the channel, and establish strong non-covalent interactions with the electron-rich MoS_2_ planes to stabilize the channels. Consequently, the resulting membranes exhibit superior stability across various aqueous environments, particularly showing excellent tolerance under highly acidic (1 M H_2_SO_4_) conditions. During harsh pressure-driven crossflow operations, the membranes demonstrate fast water permeation while maintaining high rejection (> 90%) and selectivity for heavy metal ions in acidic wastewater. This strategy of leveraging multifunctional intercalants offers critical insights for the design of task-specific 2D membranes for demanding applications.

## Abstract (web summary)

Designing two-dimensional membranes to achieve tailored channels while ensuring stability remains challenging. Here, the authors use multifunctional intercalants to create stable, selective subnanochannels in MoS_2_ membranes, demonstrating their potential in wastewater treatment.

## Introduction

Membranes featuring selective water and ion transport channels have emerged as a critical focus in materials science, with far-reaching implications for desalination, ion separation, and energy conversion^1–3^. Two-dimensional (2D) nanolaminates, constructed by stacking 2D materials, have shown great promise as nanochannel membranes for molecular and ionic separation^4–7^. The nano-to-subnano interlayer channels, with tailored width and chemistry, facilitate selective mass transport through the nanoconfinement effect^8,9^. However, many 2D membranes, particularly graphene oxide (GO) membranes^10,11^, suffer from uncontrollable channel width and structural instability, severely limiting their practical applications. In contrast, 2D membranes constructed from transition metal dichalcogenides, especially MoS_2_, offer enhanced stability due to the balance of van der Waals and hydration forces^12,13^. These membranes establish effective mass transport channels by hosting water molecules^12^ or cations^14^ as “spacers” between stacked MoS_2_ nanosheets. Without these “spacers”, MoS_2_ nanosheets tend to restack into a quasi-perfect bulk state, forming diminutive channels (< 0.2 Å) and impermeable membranes^12,15^. Nevertheless, these “spacers” exhibit instability under certain conditions. For instance, the confined water can easily escape upon dehydration, causing an irreversible contraction of these channels^12^. This transient hydration-dependent structure poses significant challenges for the practical implementation of MoS_2_ membranes. In addition, the channel width of MoS_2_ membranes can dramatically shrink from 5.2 to 0.2 Å in response to a pH shift from alkaline to acidic conditions, a change attributed to the exchange of cations within the channels^14^. Despite the reversible shrinkage, the use of these membranes in acidic conditions, a field largely unexplored for MoS_2_ membranes, remains a formidable challenge. Recent efforts to address these challenges have involved the covalent grafting of functional groups onto MoS_2_ nanosheets to develop selective channels in MoS_2_ membranes^15–18^. However, these functional groups primarily act as “spacers” to define the channel width, with their ability to tailor channel chemistry and enhance structural stability remaining largely elusive. In addition, these covalent strategies can disrupt channel alignment probably due to the spatially fixed nature of covalent bonds^18^ and involve time-consuming, less sustainable processes. These issues have led us to explore channel functionalization through a non-covalent approach^19^ using multifunctional intercalants that go beyond merely serving as “spacers”. This aims to achieve tailored channel width and chemistry while enhancing structural stability of MoS_2_ membranes for their potential real-world applications.

**Fig. 1:**
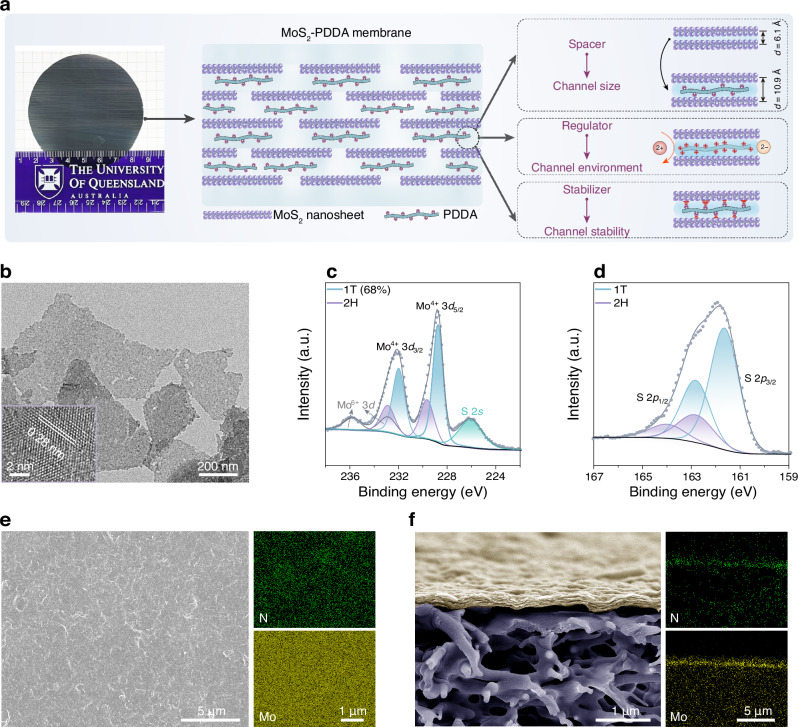
Preparation of MoS_2_-PDDA membranes. **a** Schematic illustration of the structure of MoS_2_-PDDA membranes with emphasis on the multifunctional roles of the intercalant. A photograph of a large-area (40.5 cm^2^) MoS_2_-PDDA membrane is shown in the left panel. **b** TEM image of MoS_2_ nanosheets. The inset shows a high-resolution TEM image displaying a lattice spacing of 0.28 nm corresponding to the (100) plane. **c**–**d** High-resolution XPS spectra of MoS_2_ nanosheets: Mo 3*d* (**c**) and S 2*p* (**d**). **e**–**f** Surface (**e**) and cross-sectional (**f**) morphologies of MoS_2_-PDDA membranes and the corresponding EDX mappings of N and Mo.

Herein, we present a facile, non-covalent intercalation strategy to engineer selective, highly stable subnanochannels in MoS_2_ membranes, targeting acidic wastewater treatment under harsh conditions. This approach employs polymeric quaternary ammoniums (QAs), specifically poly(diallyldimethylammonium chloride) (PDDA), as a multifunctional intercalant within MoS_2_ membranes. This method involves co-assembling MoS_2_ nanosheets with PDDA through vacuum filtration, resulting in physically confined PDDA within the interlayer galleries (hereafter referred to as MoS_2_-PDDA membranes). Remarkably, these confined QAs play multiple critical roles: first, they act as “spacers”, effectively suppressing the restacking of MoS_2_ layers to define the channel width at ~5 Å; second, they serve as “regulators”, in which positive charges transform the channel microenvironment from negative to positive to regulate ion transport properties; third, they function as “stabilizers” to interlock MoS_2_ channels through strong non-covalent interactions, ensuring high stability across various aqueous media, including pure water, salt solutions, and even strong acid (1 M H_2_SO_4_) (Fig. 1a). As a result, the multifaceted roles of PDDA collectively contribute to exceptional separation performance and stability of MoS_2_-PDDA membranes in acidic wastewater treatment. This work represents a significant advancement in the development of task-specific 2D membranes for real-world applications, particularly in challenging environments such as industrial wastewater treatment and acid mine drainage remediation.

## Results

### Preparation of membranes

The development of MoS_2_-PDDA membranes involves a straightforward one-step process: the co-assembly of MoS_2_ nanosheets with PDDA intercalants into functional membranes via vacuum filtration. Chemical exfoliation yielded MoS_2_ nanosheets with a few-layer structure and an average lateral size of 450 nm (Supplementary Fig. 1). Transmission electron microscopy (TEM) reveals smooth, ultrathin, highly crystalline nanosheets with a lattice spacing of 0.28 nm, corresponding to the (100) plane (Fig. 1b). Generally, the exfoliation process favours MoS_2_ phase transformation from 2H to 1 T^20–22^. X-ray photoelectron spectroscopy (XPS) analysis confirms the predominance of the 1 T phase (Fig. 1c, d), further corroborated by a featureless UV–Vis spectrum (Supplementary Fig. 2). It is noteworthy that the spontaneous phase transition of 1T-MoS_2_ nanosheets back to the 2H form progresses at a relatively slow rate under ambient conditions (Supplementary Fig. 3). The electron-rich surface of 1T-MoS_2_ (around 0.25 electrons per Mo atom), attributed to electron doping during lithiation^23,24^, potentially facilitates strong affinity with positively charged species.

To fabricate the membranes, we mixed MoS_2_ nanosheets with PDDA at varying concentrations. This yielded stable binary dispersions, with tunable zeta potentials that transitioned from negative to positive (Supplementary Fig. 4). Notably, these dispersions exhibit remarkable stability, with no discernible agglomeration even after 60 days of storage. Subsequently, these dispersions were assembled into MoS_2_-PDDA membranes, where linear, flexible, and elongated PDDA chains were physically intercalated within the interlayer channels of the orderly stacked MoS_2_ nanosheets (Fig. 1a). By adjusting the PDDA concentration, we achieved precise control over the PDDA content within the resulting MoS_2_-PDDA membranes, ranging from 9.6 to 32.9 wt.% (Supplementary Fig. 5). Scanning electron microscopy (SEM) reveals that MoS_2_-PDDA membranes with 12 wt.% PDDA displayed a smooth, defect-free surface (Fig. 1e and Supplementary Fig. 6) and a compact, lamellar cross-section morphology (Fig. 1f and Supplementary Fig. 7). Energy-dispersive X-ray (EDX) mapping confirms the homogeneous distribution of PDDA within the MoS_2_ channels. While increasing PDDA content has a negligible influence on the surface morphology, it significantly affects membrane thickness and cross-section structure (Supplementary Figs. 6 and 7). Excessively high PDDA content like 32.9 wt.% appears to disrupt the regular stacking of MoS_2_ nanosheets. Moreover, membrane thickness exhibits a near-linear correlation with the filtered dispersion volume, enabling precise thickness control over the MoS_2_-PDDA membranes (Supplementary Fig. 8).

### Characterization of interlayer channels

**Fig. 2:**
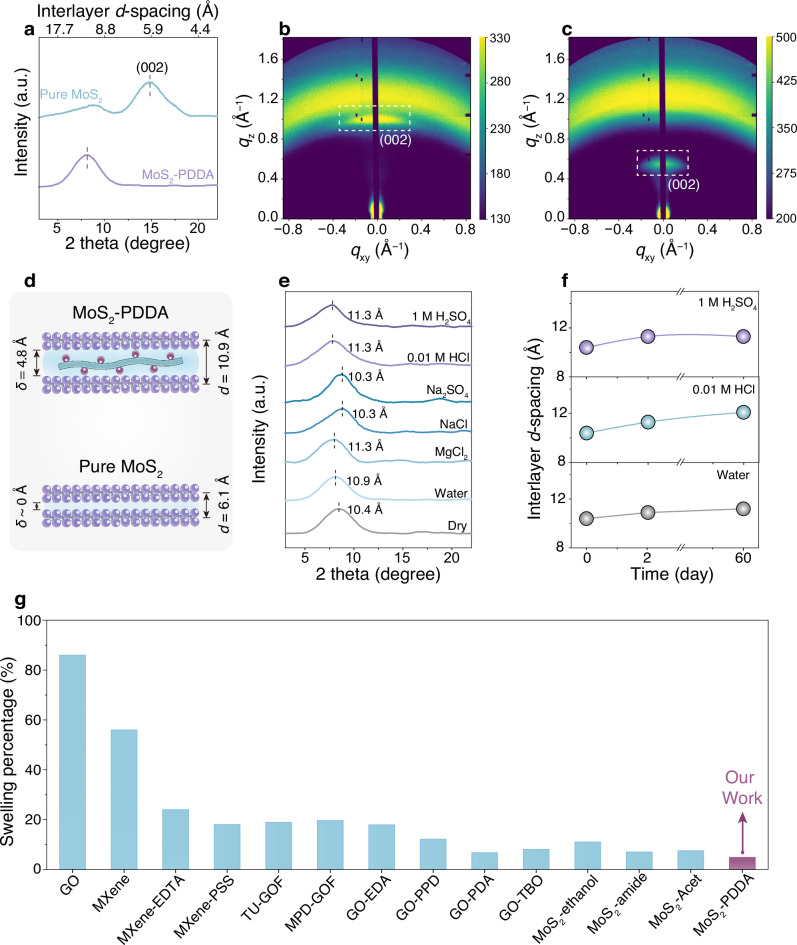
Structural characterization and stability of interlayer channels. **a** XRD patterns of pure MoS_2_ and MoS_2_-PDDA membranes in the wet state. **b**–**c** GISAXS images of pure MoS_2_ (**b**) and MoS_2_-PDDA (**c**) membranes. **d** Schematic illustration of the developed channel in pure MoS_2_ and MoS_2_-PDDA membranes. **e** XRD patterns of MoS_2_-PDDA membranes in various aqueous media. **f** Evolution of the interlayer *d*-spacing of the MoS_2_-PDDA membrane at varied pH levels represented by pure water, 0.01 M HCl, and 1 M H_2_SO_4_ over an extended period of immersion. **g** Comparison of the swelling percentage between the MoS_2_-PDDA membrane and other 2D membranes reported in the literature. Note that, unless otherwise specified, the PDDA content in MoS_2_-PDDA membranes is 12.0 wt.%.

To investigate the microstructure of MoS_2_-PDDA membranes, we conducted X-ray diffraction (XRD) and grazing-incidence small-angle X-ray scattering (GISAXS) analysis. As shown in Fig. 2a, pure MoS_2_ membranes display a prominent (002) diffraction peak at 2*θ* = 14.8°, corresponding to an interlayer *d*-spacing of 6.0 Å. A broad lower-angle peak attributed to the (001) plane because of the adsorbed Li^+^ ion on the 1T-MoS_2_ region, is also observed, consistent with previous reports^14,25^. In contrast, PDDA incorporation (12.0 wt.%) into MoS_2_ membranes shifts the (002) peak to 2*θ* = 8.1°, expanding the *d*-spacing to 10.9 Å. Further investigation of PDDA content variation (9.6–21.7 wt.%) reveals a subtle influence on the *d*-spacing (Supplementary Fig. 9). Correlation of membrane thickness with *d*-spacings indicates that insufficient PDDA content results in non-expanded channels, while excessive PDDA loading leads to over-expanded counterparts (Supplementary Fig. 10). GISAXS measurement reveals that PDDA intercalation causes a downward shift in the arc from the diffraction projection signal of the (002) plane, from *q*_z_ = 1.04 to 0.59 Å^–1^, indicating an expanded *d*-spacing (Fig. 2b, c and Supplementary Fig. 11). This finding aligns well with the XRD results. Moreover, the arc becomes more concentrated, suggesting improved stacking order of MoS_2_ nanosheets within the membrane (Schematic illustration in Supplementary Fig. 12). These findings suggest that the non-covalent intercalation of PDDA effectively preserves the long-range order of MoS_2_ channels, different from the observed stacking disorder caused by covalent functionalization^18^. By subtracting the thickness of a MoS_2_ monolayer (~6 Å)^15,18^ from the obtained *d*-spacings, we estimated the effective channel width (*δ*) of MoS_2_-PDDA membranes to be 4.8 Å in the wet state, an ideal dimension for water–ion selectivity. In contrast, pure MoS_2_ membranes possesses diminutive channels (*δ* < 0.2 Å) that are expected to be impermeable to water (Fig. 2d).

Given the critical importance of size-fixed channels for real-world applications, we investigated the anti-swelling properties of MoS_2_-PDDA membranes. Membranes with lower PDDA content (9.6–16.7 wt.%) exhibit excellent structural stability and minimal swelling upon an extended water immersion over 60 days (Supplementary Figs. 13 and 14). By contrast, excessive PDDA intercalation (21.7 and 32.9 wt.%) leads to uncontrollable delamination. We further explored the anti-swelling stability of MoS_2_-PDDA membranes with 12.0 wt.% PDDA intercalation. Notably, they exhibit a consistent *d*-spacing with variations of less than 1 Å across various environments (Fig. 2e). Even in a highly acidic environment (1 M H_2_SO_4_), the membrane shows remarkable non-swelling stability over a 60-day period, substantiating their exceptional acid tolerance (Fig. 2f). The swelling percentage is merely 4.8% in water, surpassing most reported 2D membranes including covalently functionalized MoS_2_ membranes^17^ (Fig. 2g and Supplementary Table 1). These findings highlight the potential of our MoS_2_-PDDA membranes with non-swelling, acid-tolerant subnanochannels for practical water treatment, particularly in challenging acidic environments where many existing membranes fall short.

### Chemical microenvironments of interlayer channels

**Fig. 3:**
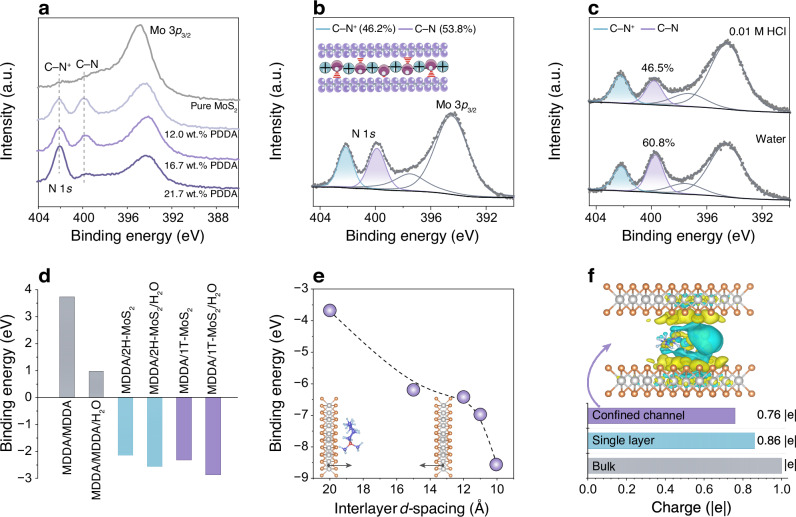
Chemical microenvironments and in-channel interactions in MoS_2_-PDDA membranes. **a** High-resolution N 1*s* XPS spectra of MoS_2_-PDDA membranes with different PDDA contents. **b** The composition percentage of C–N^+^ and C–N in the MoS_2_-PDDA membrane (12.0 wt.% PDDA) obtained from the deconvoluted results of the high-resolution N 1*s* XPS spectrum. The inset illustrates the dual chemical environment of QAs within MoS_2_ channels. **c** High-resolution N 1*s* XPS spectra of the MoS_2_-PDDA membrane (12.0 wt.% PDDA) following exposure to water and acid (0.01 M HCl) for 30 days. **d** Binding energies between MDDA/MDDA, MDDA/1T-MoS_2_, and MDDA/2H-MoS_2_ determined by DFT calculations. **e** Binding energies between MDDA/1T-MoS_2_ under nanoconfinement as a function of interlayer *d*-spacing. The inset depicts two parallel 1T-MoS_2_ monolayers with tunable interlayer *d*-spacing, where MDDA is positioned adjacent to one monolayer plane. **f** Calculated charges of MDDA under different conditions: in bulk, in interaction with a 1T-MoS_2_ monolayer, and confined within a 1T-MoS_2_ channel featuring a *d*-spacing of 10 Å. The upper panel displays the charge density differences for MDDA/1T-MoS_2_ under nanoconfinement, with yellow and blue areas representing charge accumulation and charge depletion, respectively.

The exceptional stability of MoS_2_-PDDA membranes in aqueous solutions, despite the absence of covalent crosslinking, prompts an in-depth investigation into the interfacial interactions within the nanoconfined channels. To probe the channel microenvironment, XPS analysis was conducted on MoS_2_-PDDA membranes with varying PDDA content. As shown in Fig. 3a, the peak centred at 402.1 eV is ascribed to the inherent C–N^+^ from the cationic QA moieties (for comparison, see the N 1*s* XPS spectrum of pure PDDA in Supplementary Fig. 15). Interestingly, an additional peak appears at 399.9 eV, which diminishes with the increase of PDDA content. This new peak can be ascribed to the partially neutralized C–N, due to electron density shifts from electron-rich 1T-MoS_2_ to the positively charged C–N^+^ under nanoconfinement. This electron density redistribution signals the existence of favourable interactions between the charged C–N^+^ and the 1T-MoS_2_ surface, which help to anchor the QA moieties to the 1T-MoS_2_ plane, simultaneously promoting the interlocking of adjacent MoS_2_ layers. Thus, the proportion of C–N serves as an indicator of this interlocking degree. A high PDDA loading (21.7 wt.%) exhibits a relatively low fraction of C–N within MoS_2_-PDDA membranes, well explaining their poor structural stability in aqueous solution (Supplementary Fig. 13). At an appropriate PDDA content (12.0 wt.%), the C–N percentage reaches 53.8% (Fig. 3b), corresponding to robust interlocking effect and superior structural stability. Concurrently, the percentage of 1T-MoS_2_ reduces to 41% (Supplementary Fig. 16). This interlocking effectively suppresses channel expansion and enhances the PDDA confinement. Furthermore, there are still abundant residual C–N^+^ sites, which impart positive charges to the channel and shift the surface zeta potential from negative to positive (Supplementary Fig. 17). More importantly, this dual chemical environment of QAs within MoS_2_ channels persists after 30-day immersion in both water and acid (Fig. 3c), as suggested by the well-maintained C–N/C–N^+^ ratio. The slight decrease in the C–N proportion following acid exposure may be attributed to competition between protons and QAs for interaction with 1T-MoS_2_, as protonation of 1T-MoS_2_ can lead to the formation of covalent S–H bonds^14,26^.

We further performed density functional theory (DFT) calculations to elucidate molecular-level interactions. For simplicity, it should be noted that PDDA is modelled as its monomeric repeating units (referred to as MDDA). The binding energies between MDDA/MDDA, MDDA/1T-MoS_2_, and MDDA/2H-MoS_2_, were first calculated respectively in a simplified one-on-one configuration, where a single MDDA molecule was stabilized near the surface of MoS_2_ planes (Fig. 3d and Supplementary Fig. 18). MDDA exhibits a repulsive interaction with itself, favouring a dispersed configuration within the channels. Conversely, a strong attractive interaction is observed between MDDA and MoS_2_, with 1T-MoS_2_ exhibiting a higher affinity for MDDA compared to 2H-MoS_2_. To highlight the nanoconfinement effect, we applied a one-between-two mode, positioning the MDDA molecule between two parallel 1T-MoS_2_ monolayers with adjustable interlayer *d*-spacing (Fig. 3e). The binding energy increases as the *d*-spacing decreases, particularly at *d*-spacing below 12 Å, consistent with our XRD observations. This underscores the effectiveness of the nanoconfinement effect in enhancing the structural stability of MoS_2_-PDDA membranes. Charge density distribution analysis in the nanoconfined space reveals considerable charge accumulation at the MDDA/1T-MoS_2_ interface (Fig. 3f). Bader charge analysis shows that the net charge of MDDA reduces to 0.76 |e|, indicating enhanced charge transfer compared to the non-confined case. This charge redistribution signifies an electron density shift from 1T-MoS_2_ to MDDA, governing the favourable interaction between MoS_2_ nanosheets and PDDA in MoS_2_-PDDA membranes.

### Water and ion transport properties

Understanding the transport properties of water and ions across MoS_2_-PDDA membranes is crucial for evaluating their potential in water treatment applications. We assessed the separation performance of MoS_2_-PDDA membranes with varying PDDA contents using single MgCl_2_ solutions as the feed (Supplementary Fig. 19). Pure MoS_2_ membranes exhibit negligible water permeability due to their diminutive channel dimensions (Fig. 2d)^12,15^. The intercalation of PDDA as a “spacer” initially increases water permeance with the increase of PDDA content, and then plateaus at higher loadings. This behaviour can be attributed to the expansion of the channel width of ~ 5.2 Å in MgCl_2_ solutions (Fig. 2e). With a channel width larger than water molecules but smaller than hydrated Mg^2+^ ions (Supplementary Table 2), the membrane demonstrates high MgCl_2_ rejection. However, increasing PDDA content leads to a decrease in MgCl_2_ rejection, likely due to the excess PDDA hindering the formation of well-aligned 2D channels. Thus, the optimized PDDA content within MoS_2_ channels is determined as 12 wt.%. Subsequently, investigation of membrane thickness reveals that increased thickness results in reduced water permeance yet increased MgCl_2_ rejection, attributed to enhanced mass transfer resistance and simultaneous minimized defects^27,28^(Supplementary Fig. 20). With a thickness of 160 nm, the MoS_2_-PDDA membrane displays a MgCl_2_ rejection of 91.4% and a water permeance of 11.4 L m^–2^ h^–1^ bar^–1^, positioning it as the optimal candidate for subsequent study and optimization.

**Fig. 4:**
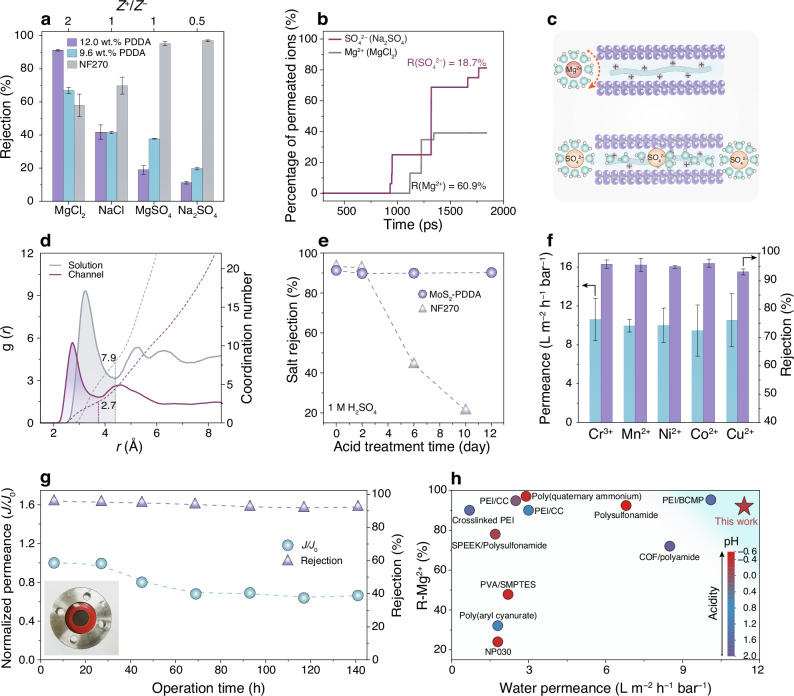
Water and ion transport properties, wastewater treatment performance, and operational stability of MoS_2_-PDDA membranes. **a** Comparison of MoS_2_-PDDA membranes (9.6 wt.% and 12.0 wt.% PDDA) and commercial nanofiltration membranes (i.e., NF270) in their rejection for the salts with different *Z*^+^/*Z*^–^. **b** Percentages of permeated Mg^2+^ (in MgCl_2_) and SO_4_^2–^ (in Na_2_SO_4_) ions through a simulated channel of MoS_2_-PDDA membranes as a function of simulation time from MD simulations. **c** Schematic illustration of the repulsion of Mg^2+^ ions and the dehydration of SO_4_^2−^ ions. **d** RDF of water-O around SO_4_^2−^ and the corresponding coordination numbers of water molecules in both the feed solution and the membrane channels. **e** Salt rejection of MoS_2_-PDDA membranes and commercial nanofiltration membranes (i.e., NF270) as a function of their exposure in 1 M H_2_SO_4_ for different time periods. **f** Separation performance of MoS_2_-PDDA membranes in treating acidic wastewater containing heavy metal ions. **g** Long-term stability of MoS_2_-PDDA membranes under the crossflow operation. *J*_0_ and *J* denote the water permeance at 6 h and beyond 6 h, respectively, during the 141-h operation. The insert shows the photograph of the membrane in the module after testing. **h** Comparative analysis of the separation performance and acid stability of MoS_2_-PDDA membranes against previously reported acid-tolerant membranes. Note that, unless otherwise specified, the PDDA content in MoS_2_-PDDA membranes is 12.0 wt.%. R-Mg^2+^ represents the rejection for MgCl_2_. The error bars in this figure indicate the standard deviations of data obtained from at least three independent tests.

Unlike commercial nanofiltration membranes (e.g., NF270, DuPont), MoS_2_-PDDA membranes (12.0 wt.% PDDA) exhibit a unique salt rejection order: MgCl_2_ (91.4%) > NaCl (41.8%) > MgSO_4_ (18.9%) > Na_2_SO_4_ (11.2%), highly influenced by the cation-to-anion valence ratio (*Z*^+^/*Z*^–^) of the salt (Fig. 4a). Given the larger diameter of the hydrated ions relative to the channel width (Supplementary Table 2), the observed ion transport behaviour cannot be solely explained by size sieving. Instead, charge effects including ion–water (i.e., ion hydration), ion–channel, and ion–ion interactions should be taken into account. Notably, the hydration shells surrounding the ions, which define their effective size, can be partially removed or rearranged to allow the passage of ions through narrow channels when favourable ion–channel interactions compensate for the energy cost of dehydration^2,29^. In our case, the electrostatic attraction between anions (e.g., SO_4_^2–^) and positively charged QAs at the channel entrance is expected to facilitate ion dehydration and entry, while cations (e.g., Mg^2+^) will be repelled and are less likely to dehydrate and enter. Furthermore, the pairing between anions and cations to maintain charge neutralization, specifically the balance between the force repelling cations and the force attracting anions, determines the rejection for the salts. Since these forces depend on the ion valence, salt rejection shows a strong dependency on *Z*^+^/*Z*^–^ (refs. ^30,31^), that is, the salt with higher *Z*^+^/*Z*^–^ shows higher rejection and vice versa. To highlight the charge effects, we reduced the PDDA loading (9.6 wt.%) to decrease the positive charge density while maintaining the channel size (Supplementary Fig. 9). As expected, the salt rejection of this membrane becomes less dependent on *Z*^+^/*Z*^–^, yielding declined MgCl_2_ rejection (66.9%) but enhanced rejection against MgSO_4_ (37.8%) and Na_2_SO_4_ (19.8%), due to weakened ion–channel interactions. Similarly, increasing feed concentration decreases MgCl_2_ rejection (Supplementary Fig. 21), as higher ionic strength effectively screens the charge, weakening ion–channel interactions. These observations confirm the crucial role of positive charges in the subnanochannels for the high rejection of multivalent cations.

To gain further insights into the ion transport behaviour at the molecular level, molecular dynamics (MD) simulations were conducted on a slit-like channel model. The simulation system comprises of two horizontally aligned 1T-MoS_2_ monolayers with a *d*-spacing of 11 Å, incorporated with MDDA to mirror the channel microenvironment of MoS_2_-PDDA membranes. The simulated salt rejection order aligns well with our experimental findings (Supplementary Fig. 22), similarly showing a notable difference in the rejection rates of MgCl_2_ and Na_2_SO_4_ (Fig. 4b). Despite the large diameter of SO_4_^2–^ ions compared to the channel width, the low rejection of Na_2_SO_4_ suggests significant dehydration of SO_4_^2–^ upon entry into the channel, whereas Mg^2+^ ions undergo repulsion and are difficult to dehydrate and enter (Schematic illustration in Fig. 4c). Radial distribution function (RDF) analysis of water-O around SO_4_^2−^ confirms a reduction in the number of water molecules from 7.9 to 2.7 within its inner hydration shells, as indicated by weakened and shifted peaks (Fig. 4d). This dehydration is energetically compensated by the favourable attraction between SO_4_^2−^ and positively charged QAs in the channel. These observations collectively demonstrate the significant dehydration of high-valence counter-ions as they enter and diffuse within the positively charged channels built in our study.

### Acid-tolerant stability and acidic wastewater treatment

As discussed, we have developed a straightforward non-covalent intercalation strategy that employs PDDA as a potent intercalant to functionalize MoS₂ membranes effectively. Unlike reported intercalants with limited functions, the intercalated PDDA performs multiple roles (Supplementary Table 3). First, PDDA acts as “spacers”, preventing the restacking of MoS_2_ nanosheets and defining the channel width of ~5 Å without disrupting the channel order, which is ideal for fast and selective water permeation. Second, PDDA serves as “regulators”, in which the cationic QA moieties within the channels transform the channel microenvironment from negative to positive, enabling regulated ion transport. Third, PDDA functions as “stabilizers” to interlock channels through strong non-covalent interactions, ensuring high stability of the resulting membrane across various aqueous media, particularly excellent acid tolerance under extremely acidic (1 M H_2_SO_4_) conditions. These characteristics have led us to assess the potential of MoS_2_-PDDA membranes for the treatment of acidic wastewater containing heavy metal ions.

We first evaluated the separation performance of MoS_2_-PDDA membranes at different pH levels and found that shifting the feed solution pH from 6.5 to 2.0 enhances water permeance with a negligible decrease in MgCl_2_ rejection (Supplementary Fig. 23). Then we examined acid tolerance of the membranes by monitoring the changes in their separation performance after exposure to 1 M H_2_SO_4_ over different durations. The MoS_2_-PDDA membranes display satisfactory acid-tolerant stability, as evidenced by the maintained MgCl_2_ rejection during prolonged acid exposure. In contrast, benchmark nanofiltration membranes (e.g., NF270) suffer severe performance degradation, due to the susceptibility of their polyamide chemistry to hydrolysis^32,33^ (Fig. 4e). Encouraged by this acid tolerance, we further explored the effectiveness of MoS_2_-PDDA membranes in treating acidic wastewater. In a continuous crossflow using simulated acidic wastewater (pH 2.0), the membrane exhibits high removal rates for various heavy metal ions, including Cr^3+^ (95.9%), Mn^2+^ (95.7%), Ni^2+^ (95.0%), Co^2+^ (96.2%), and Cu^2+^ (93.2%), coupled with a stable water permeance of 10 L m^–2^ h^–1^ bar^–1^ (Fig. 4f). Meanwhile, the membrane shows satisfactory antifouling properties, achieving a high flux recovery of 80% under acidic conditions (pH 2.0) when treating practical water from the Brisbane River (Supplementary Fig. 24). Operational stability, crucial for real-world applications, was further evaluated under continuous crossflow conditions, where the shear force may pose risks for the integrity of 2D membranes^34^. In such a challenging testing condition, the membrane demonstrates remarkable stability, with minimal performance degradation over 720 min (Supplementary Fig. 25) and less than 30% decrease in water permeance during a 141-h test (Fig. 4g). This decrease can be attributed to the compaction of MoS_2_ channels under long-term high-pressure conditions, as evidenced by the slight decline in the pure water flux at higher pressure (Supplementary Fig. 26). However, this compaction does not compromise the membrane rejection performance, maintaining a rejection of MgCl_2_ above 90% during this period. The membrane still maintains its structural integrity without visible delamination after long-term testing.

The separation performance, along with the acid tolerance, of the MoS_2_-PDDA membranes was compared with those of previously reported membranes. Our membranes demonstrate comparable separation performance with the state-of-the-art novel membranes including GO, MXene, MoS_2_, metal-organic framework (MOF), and covalent-organic framework (COF) membranes, and benchmark commercial nanofiltration membranes (Supplementary Fig. 27 and Supplementary Table 4). Notably, our membranes exhibit superior acid-tolerant stability, outperforming most of reported pH-resistant membranes (Fig. 4h and Supplementary Table 5). The combination of high selectivity, excellent stability, and sustained performance in acidic environments makes them highly competitive for acid-tolerant wastewater treatment under harsh conditions.

## Discussion

We report the employment of multifunctional intercalants (i.e., PDDA) for engineering selective, stable subnanochannels in MoS_2_ membranes for wastewater treatment under harsh conditions. The intercalated PDDA in MoS_2_-PDDA membranes functions as a three-in-one intercalant, integrating the roles of “spacers”, “regulators”, and “stabilizers” simultaneously. First, PDDA acts as “spacers”, preventing the restacking of MoS_2_ nanosheets and defining the channel width at ~5 Å without disrupting the channel order, which is ideal for fast and selective water permeation. Second, PDDA serves as “regulators”, in which the inherently positively charged QA moieties transform the microenvironment from negative to positive, enabling selective ion transport enabled by the interplay of ion–water, ion–ion, and ion–channel interactions. Third, PDDA functions as “stabilizers” to interlock MoS_2_ channels through strong non-covalent interactions, ensuring high stability of the resulting membrane across various aqueous media, including pure water, salt solutions, and even strong acid (1 M H_2_SO_4_). The multifaceted roles of PDDA contribute synergistically to the exceptional separation performance and stability of MoS_2_-PDDA membranes in acidic wastewater treatment. This multifunctionality represents a feature not demonstrated by traditional intercalants reported in the existing literature. Importantly, the multifunctionality of intercalants is theoretically broad and holds potential for application to other transition metal dichalcogenides and QA-bearing molecules. Overall, the development of multifunctional intercalants for functionalizing 2D membranes provides critical insights into the design of task-specific membranes for demanding applications, advancing our understanding of the functionalization–structure–performance relationship in 2D membranes.

## Methods

### Chemical exfoliation of MoS_2_ nanosheets

MoS_2_ nanosheets were prepared via lithium intercalation^22^, as described below. First, 1 g of MoS_2_ bulk powder was mixed with 10 mL of an n-butyllithium hexane solution, followed by stirring in a nitrogen atmosphere at room temperature for 48 h. Next, the obtained MoS_2_ colloidal suspension was filtered and then washed with hexane several times. Subsequently, the lithium-intercalated MoS_2_ (Li_x_MoS_2_) powder was exfoliated in deionized water via sonication for 1 h. Finally, the solution was centrifugated at 10,000 rpm and 5000 rpm to remove lithium ions and unexfoliated particles, respectively. The as-synthesized MoS_2_ nanosheets with a concentration of 0.25 mg mL^–1^ were stored at 4 °C.

### Fabrication of MoS_2_-PDDA membranes

First, PDDA solution with different concentrations was mixed with the diluted solution of MoS_2_ nanosheets, forming a binary dispersion with MoS_2_ concentration of 10 mg L^–1^ and varied PDDA concentration (0–10 mg L^–1^). Then, the as-prepared MoS_2_-PDDA dispersion was vacuum-filtrated through a porous PES substrate with nominal pore size of 100 nm, allowing the co-assembly of MoS_2_ nanosheets and PDDA. MoS_2_-PDDA membranes were finally obtained after drying under vacuum at 40 °C for 4 h. Membrane thickness can be easily tuned by altering the filtration volume of MoS_2_-PDDA mixture.

### Characterizations

The morphology of prepared MoS_2_ nanosheets was observed using field emission TEM (JEM-2100F, JEOL, Japan). The thickness and lateral size of MoS_2_ nanosheets were determined using atomic force microscopy (AFM, Dimension Icon, Bruker, USA) operated in tapping mode. A UV–Vis spectrophotometer (UV1800, Shimadzu, Japan) was employed to detect the UV–Vis absorption of MoS_2_ nanosheets in the aqueous solution. Zeta potentials of MoS_2_-PDDA mixture were measured using a Zetasizer (Zetasizer Nano, Malvern Instruments, UK). The surface and cross-sectional morphologies of prepared MoS_2_ membranes were investigated using field emission SEM (GeminiSEM 500, ZEISS, Germany), and the elemental distribution was detected using the equipped EDX spectrometer (Aztec X-Max Extreme, Oxford Instruments, UK). XRD patterns of prepared MoS_2_ membranes were recorded using an X-ray diffractometer (TTR-III, Rigaku, Japan) with Cu Kα radiation (40 kV, 200 mA) in the 2-theta range of 3–25° at a step size of 0.02° and a recording rate of 0.15 s. GISAXS measurement was performed on a Xeuss 2.0 system (XENOCS, France), irradiated at a grazing angle of 0.12°. XPS (Kratos Axis Supra+, Japan) with a monochromatized Al Kα X-ray source at 1486.6 eV was used to analyse the phase of exfoliated MoS_2_ nanosheets and the surface chemistry of prepared MoS_2_ membranes. The surface zeta potentials of MoS_2_ membranes were measured by an electrokinetic analyser (SurPASS 3, Anton Paar, Austria) using 1 mM KCl as the electrolyte solution.

### Density functional theory (DFT) calculations

All the DFT calculations were performed using the generalized gradient approximation of the Perdew-Burke-Ernzerhof functional within the Vienna Ab Initio Simulation Package 6.1 (VASP)^35^. The core-valence interaction was represented by the projector augmented wave approach^36^, and the energy cutoff for the plane wave was set to 400 eV. The Brillouin zone integration was sampled with Monkhorst mesh of 2 × 2 × 1 for all the geometry optimizations. The energy and force riteria for electron density convergence were set to 10^–4^ eV and 0.05 eV Å^–1^, respectively. MoS_2_ monolayers of both 1 T and 2H phases were built with 16 Mo atoms and 32 S atoms. PDDA was represented by its monomeric repeating unit (MDDA). After optimizing the interaction configurations, the binding energies between MDDA/MDDA, MDDA/2H-MoS_2_, and MDDA/1T-MoS_2_ in a one-on-one mode was calculated. Furthermore, the interaction energies between MDDA/1T-MoS_2_ in a one-between-two mode was calculated, where MDDA is positioned between two parallel 1T-MoS_2_ monolayers with adjustable interlayer *d*-spacing from 20 to 10 Å. Taking the MDDA/1T-MoS_2_ system as an example, the binding energy was calculated using Eq. (1),

1 $$\Delta E=	 E\,\left(1{\mbox{T}}{\mbox{-}}{\mbox{Mo}}{{\mbox{S}}}_{2}/{{\mbox{H}}}_{2}{\mbox{O}}/{\mbox{MDDA}}/{{\mbox{H}}}_{2}{\mbox{O}}\right)\\ 	 -E\,\left(1{\mbox{T}}{\mbox{-}}{\mbox{Mo}}{{\mbox{S}}}_{2}/{{\mbox{H}}}_{2}{\mbox{O}}\right)-E\,\left({\mbox{MDDA}}/{{\mbox{H}}}_{2}{\mbox{O}}\right)$$

where *E* (1T-MoS_2_/H_2_O/MDDA/H_2_O) represents the total energy of a combined system of a 1T-MoS_2_ monolayer and an MDDA molecule in water, whereas *E* (1T-MoS_2_/H_2_O) and *E* (MDDA/H_2_O) represents the energy of a 1T-MoS_2_ monolayer and an MDDA molecule in water, respectively. Moreover, the Bader charge analysis was conducted for the MDDA/1T-MoS_2_ system in the one-between-two mode at a fixed interlayer *d*-spacing of 10 Å.

### Evaluation of separation performance

Separation performance of prepared MoS_2_ membranes and benchmark commercial nanofiltration membranes (e.g., NF270 and NF90) was evaluated using a laboratory-made crossflow filtration system with an effective filtration area of 2.5 cm^2^. The testing was conducted under applied pressure of 4 bar at room temperature with a crossflow rate of 80 L h^–1^. Aqueous solutions of MgCl_2_, MgSO_4_, NaCl, or Na_2_SO_4_ (1 mg mL^–1^, without pH adjustment) and simulated acidic wastewater containing MnCl_2_, NiCl_2_, CoCl_2_, CuCl_2_, or CrCl_3_ (0.1 mg mL^–1^, pH = 2) were used as the feed solution. All the membranes were compacted for 1 h to ensure a steady state before collecting the permeation solution. The water permeance (*J*, L m^–2^ h^–1^ bar^–1^) was calculated using Eq. (2),

2 $$J=\frac{V}{A\times t\times \Delta P}$$

where *V* (L) is the volume of permeated solution, *A* (m^2^) the effective membrane area, *t* (h) the permeation time, and Δ*P* (bar) the applied pressure. The salt rejection (*R*, %) was determined according to Eq. (3),

3 $$R=\left(1-\frac{{c}_{{\mbox{p}}}}{{c}_{{\mbox{f}}}}\right)\times 100\%$$

where *c*_p_ and *c*_f_ are the solute concentrations of the permeate and feed, respectively. The salt concentration of MgCl_2_, MgSO_4_, NaCl, and Na_2_SO_4_ was determined by a conductivity metre (DDBJ-350, Shanghai Leici Instrument Company, China), while the accurate metal ion concentration was quantified by inductively coupled plasma optical emission spectrometry (ICP-OES, Optima 7300 DV, PerkinElmer, USA).

## Supplementary information

Supplementary Information

Transparent Peer Review file

## Source data

Source data

## Data Availability

The data supporting the findings of this study are available in the main text and the Supplementary Information file. Raw data generated during this study are available upon request from the corresponding authors. Source data are provided with this paper.
