# Supplementary material for: Multifunctional intercalants create stable subnanochannels in MoS2 membranes for wastewater treatment
Source: Nat Commun. 2025 Sep 24;16:8353. doi: 10.1038/s41467-025-58409-x (PMC12460844; doi:10.1038/s41467-025-58409-x)
Supplement: Supplementary file 1 — Supplementary Information [file 41467_2025_58409_MOESM1_ESM.pdf]

Supplementary Information

Multifunctional intercalants create stable subnanochannels in MoS_2 membranes for wastewater treatment

Hao Zhang[1,2], Ming Yong[1,3], Ting Hu[3], Yuan Kang[3], Zhuyuan Wang[1], Zhonghao Xu[1], Xuefeng Li[1], Xin Sun[1], Lijun Guo[1,4], Fangmeng Sheng[2], Xiangkang Zeng[1], Zhikao Li[3], Xingya Li[2*], Huanting Wang[3], Tongwen Xu[2*], Xiwang Zhang[1,5*]

[1]UQ Dow Centre for Sustainable Engineering Innovation, School of Chemical Engineering, The University of Queensland, St Lucia, Queensland, 4072, Australia

[2]Key Laboratory of Precision and Intelligent Chemistry, School of Chemistry and Materials Science, University of Science and Technology of China, Hefei, 230026, P. R. China

[3]Department of Chemical & Biological Engineering, Monash University, Clayton, Victoria, 3800, Australia

[4]National Engineering Research Center of Clean Technology in Leather Industry, Sichuan University, Chengdu, 610065, P. R. China

[5]ARC Centre of Excellence for Green Electrochemical Transformation of Carbon Dioxide (GETCO2), The University of Queensland, St Lucia, Queensland, 4072, Australia

*Corresponding authors: xingyali@ustc.edu.cn (Xingya Li); twxu@ustc.edu.cn (Tongwen Xu); xiwang.zhang@uq.edu.au (Xiwang Zhang)

Supplementary Methods

Materials. MoS_2 bulk powder (particle size < 2 μm, > 99.5%), n-butyllithium (n-BuLi, 1.6 M in hexane), poly(diallyldimethylammonium chloride) (PDDA, molecular weight of 400,000 to 500,000 Da, 20 wt.% aqueous solution) were purchased from Shanghai Aladdin Reagent Company (China). n-hexane (⩾97%) and inorganic salts, including $MgCl_2$ (⩾98%), $MgSO_4$ (⩾97%), LiCl (⩾99%), NaCl (⩾99%), Na_2SO_4 (⩾99%), $CrCl_3{\cdot}6H_2O$ (⩾98%), $MnCl_2{\cdot}4H_2O$ (⩾98%), $NiCl_2{\cdot}6H_2O$ (⩾98%), $CoCl_2{\cdot}6H_2O$ (⩾98%), and $CuCl_2{\cdot}2H_2O$ (⩾98%) were provided by Sinopharm Chemical Reagents Co., Ltd. (China). Porous poly(ether sulfone) (PES) substrates with nominal pore size of 100 nm were supplied by Xinya Filter Company (China). Commercial NF270 and NF90 membranes were provided by DuPont (USA). All the chemicals were used as received. Deionized water was provided using an RO device in our own lab.

Determination of PDDA content. Since the assembly of MoS_2-PDDA membranes is carried out in a vacuum filtration device, it is inevitable to cause the penetration of PDDA during the assembly. To quantitively describe the PDDA content incorporated in MoS_2 membranes, we can estimate its concentration in both initial dispersion and vacuum filtrate by measuring the total nitrogen (TN) concentration via an established persulfate digestion method (HACH 2714100/2672245). First, we obtained the standard curve between PDDA concentration (0–6 mg L^{-1}) and corresponding TN concentration (note that the TN concentration originated from pure water was deducted). Then, we estimated the PDDA concentration of the vacuum filtrate according to the obtained linear fitting curve. Finally, assuming that there is no loss of MoS_2 nanosheets during the assembly, the PDDA content within MoS_2 membranes can be determined by the following formula,

$$\text{PDDA content (wt.\%)} = \frac{c_{\text{P0}} - c_{\text{PV}}}{c_{\text{P0}} - c_{\text{PV}} + c_{\text{M}}}$$

where c_{P0} and c_M are the concentration of PDDA and MoS_2 nanosheets in the MoS_2-PDDA suspension, respectively, while c_{PV} is the concentration of PDDA in the vacuum filtrate.

Calculation of the interlayer *d*-spacing of MoS_2 membranes. The interlayer *d*-spacing of MoS_2 membranes was estimated from the position of (002) peak obtained from the XRD patterns, according to the Bragg's law,

$$2 \times d \times \sin\theta = n \times \lambda$$

where d is the interlayer *d*-spacing, θ the incident angle, n the diffraction order (n = 1), and λ the wavelength of the X-ray (λ = 1.54 Å).

Investigation of the swelling percentage of MoS_2 Membranes. The swelling percentage of prepared MoS_2 membranes was determined according to the following equation,

$$\text{Swelling percentage} = \frac{d_{\text{im}} - d_0}{d_0} \times 100\%$$

where d_{im} and d_0 refer to the interlayer *d*-spacing of the MoS_2 membrane after and before immersion into an aqueous solution for a specified duration, respectively. The value of *d*-spacing was calculated based on Bragg's law, as previously described.

Molecular dynamics (MD) simulations. To investigate the ion transport properties of MoS_2-PDDA membranes, MD simulations were conducted using GROMACS 2019.6 (Ref.[1]). The model comprised two parallel layers of 1T-MoS_2 with an interlayer *d*-spacing of 11 Å. Given the inherent rigidity of MoS_2 layers, the atoms within the MoS_2 layers were held fixed throughout the simulations. PDDA was represented by its monomeric repeating unit (MDDA), which was uniformly dispersed within the 1T-MoS_2 channels at a loading ratio of N/Mo = 1:8, consistent with the experimental conditions (12 wt.%

PDDA in the MoS_2-PDDA membrane).

The simulation box dimensions during the solvent equilibrium process were set to 2.2 nm × 3.3 nm × 21 nm, with a designated feed region on the left side of the channel. NaCl, $MgCl_2$, Na_2SO_4, and $MgSO_4$ were randomly placed within the feed chamber at a concentration of 1 g L^{-1}, identical to the experimental value. The system was subsequently solvated with water molecules, modeled using the SPC/E water model. The Universal Force Field with QEq charges was assigned to all non-water molecules using the OBGMX tool[2].

Energy minimization was performed in multiple steps, starting with steepest-descent minimization followed by conjugate gradient minimization. Equilibration simulations were then run for 10 ns under NVT conditions, with a time step of 0.1 fs. Bond lengths involving hydrogen atoms were constrained using the LINCS algorithm[3], and the system temperature was maintained at 298.15 K using the V-rescale thermostat[4]. The Coulomb and van der Waals cutoff distances were set to 1 nm, with electrostatic interactions computed via the particle mesh Ewald method. Following equilibration, 10 ns NVT production simulations were conducted with a pulling force of 30,000 kJ mol^{-1} nm^{-2}. The ion permeation and rejection ratios were calculated as the number of cations that passed through or were retained in the channel, normalized by the initial number of ions in the feed chamber. All simulation results were analyzed and visualized using VMD software[5].

Evaluation of antifouling properties. The antifouling performance of MoS_2-PDDA membranes was assessed using practical water collected from the Brisbane River (Supplementary Figure 24a). To remove large particles and colloids, the water samples were first filtered through PES microfiltration membranes with a pore size of 100 nm (Supplementary Figure 24b and c). Initially, the membrane was stabilized at

4 bar for 0.5 h. It was then subjected to filtration with a diluted HCl solution (pH = 2) for 1 h, and the average flux during this phase was recorded as J_0. Following this, the membrane was used to filtrate the practical water (pH adjusted to 2 with HCl) for 1 h, with the flux recorded as J_f. Afterward, the fouled membrane was rinsed with pure water for 1 h, followed by filtration with the above-mentioned HCl solution for another 1 h, and the recovered flux was recorded as J_r. The flux recovery ratio (J_r/J_0) was calculated to quantify the antifouling performance.

Supplementary Figures and Tables

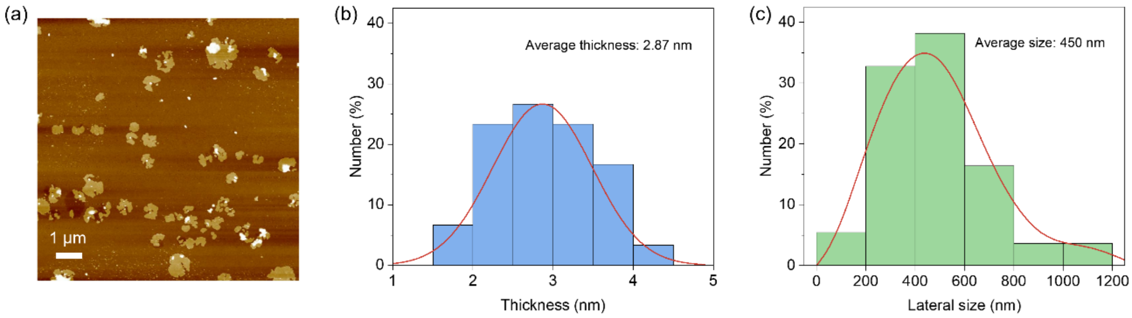

Supplementary Figure 1. (a) AFM image of exfoliated MoS_2 nanosheets and the distribution of their (b) thickness and (c) lateral size.

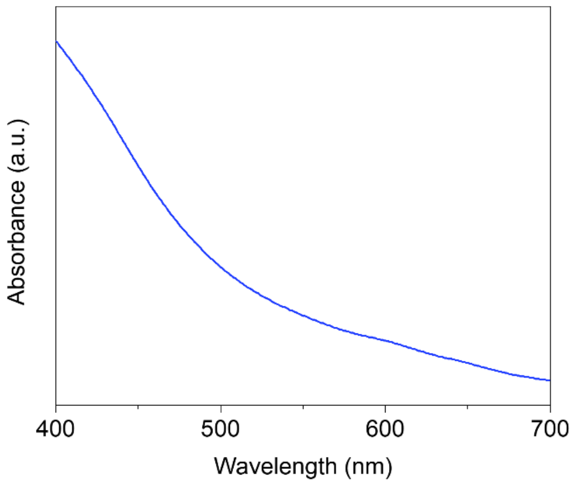

Supplementary Figure 2. UV-Vis absorption spectrum of MoS_2 nanosheets dispersed in water.

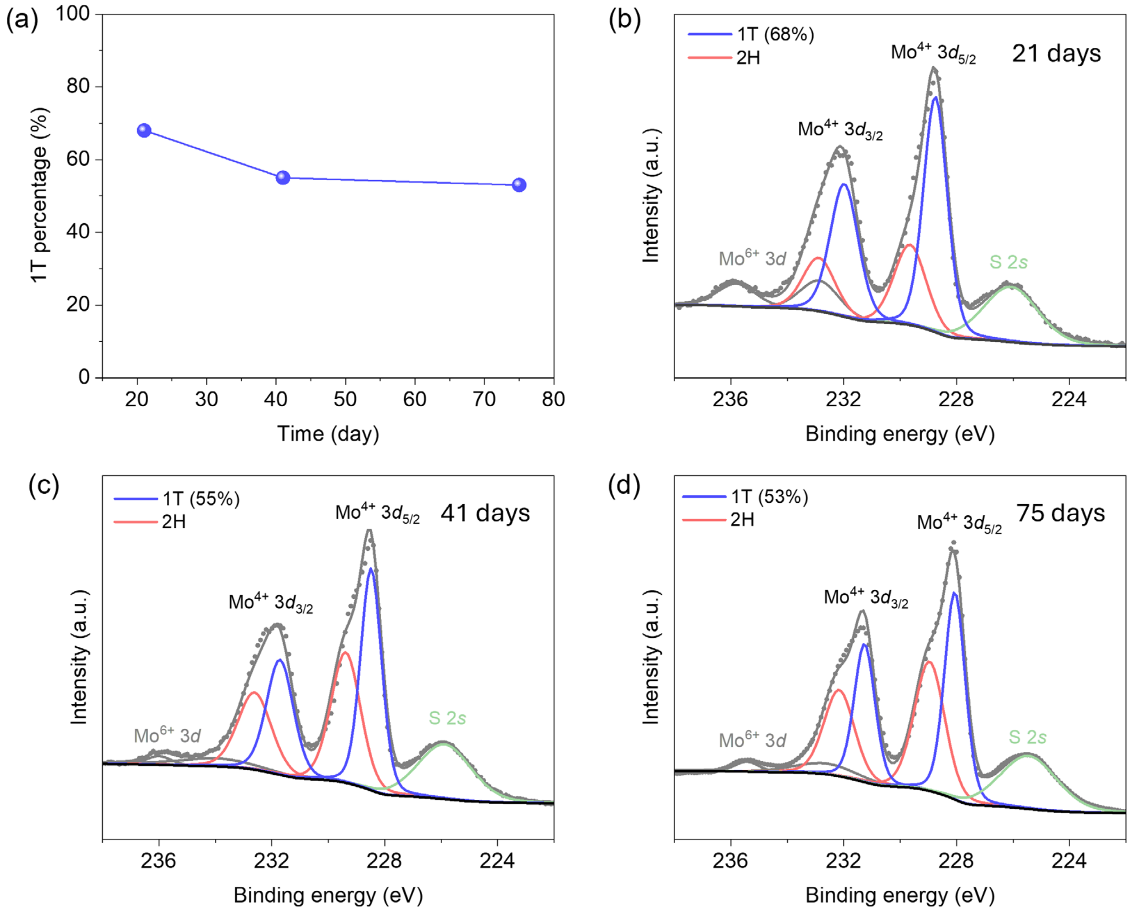

Supplementary Figure 3. Phase transition of MoS_2 nanosheets in the aqueous solution over time. (a) The percentage of 1T-MoS_2 as a function of storage time. (b–d) High-resolution Mo 3*d* XPS spectra of MoS_2 nanosheets obtained at a time duration of (b) 21, (c) 41, and (d) 75 days.

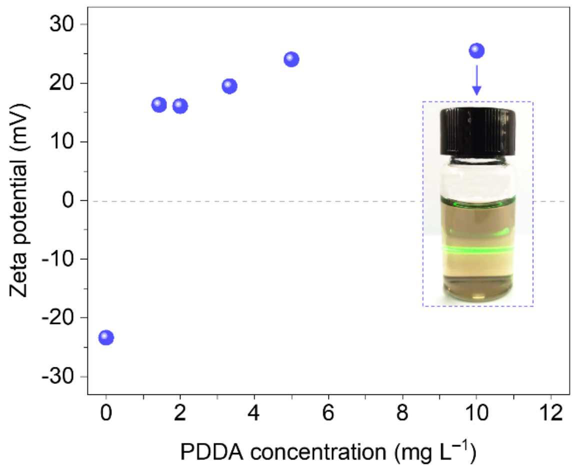

Supplementary Figure 4. Zeta-potentials of MoS_2-PDDA dispersions with different PDDA concentration. The inset shows the high stability of the dispersion with PDDA concentration of 10 mg L^{-1} after a 60-day period of storage.

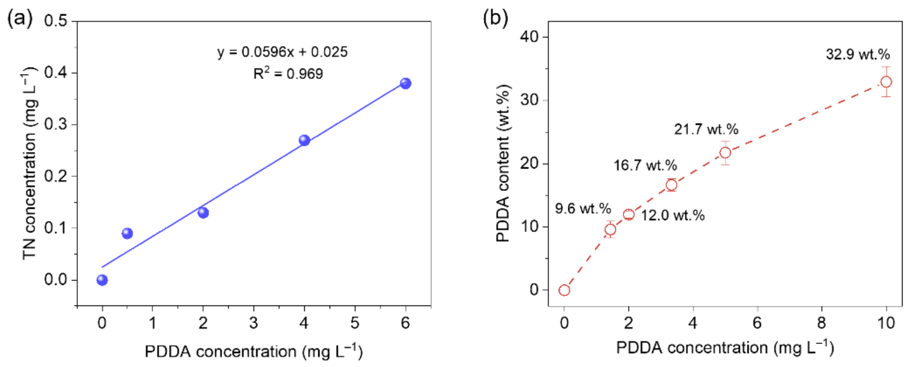

Supplementary Figure 5. (a) The fitting curve between PDDA concentration and corresponding total nitrogen (TN) concentration in the aqueous solution. (b) The relationship between PDDA concentration in the filtrated dispersion and PDDA content incorporated in MoS_2 membranes. The error bars indicate the standard deviations of the calculated PDDA content based on two independent tests.

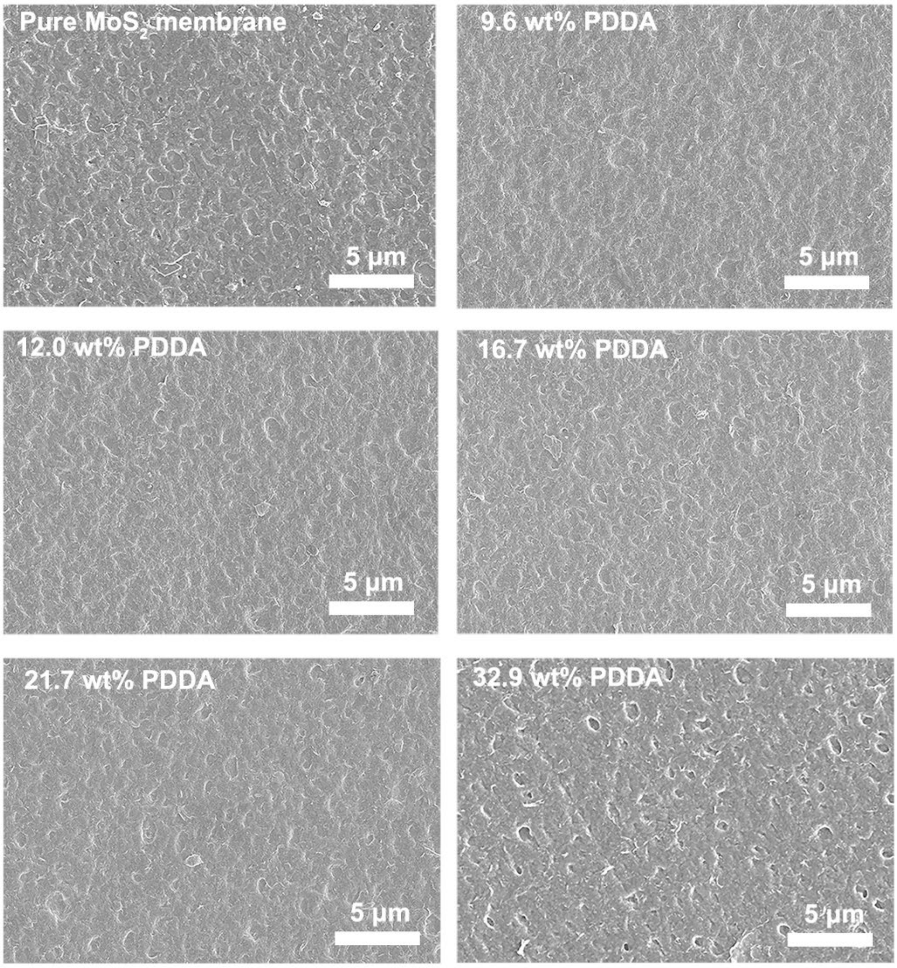

Supplementary Figure 6. Surface morphologies of MoS_2-PDDA membranes with different PDDA contents.

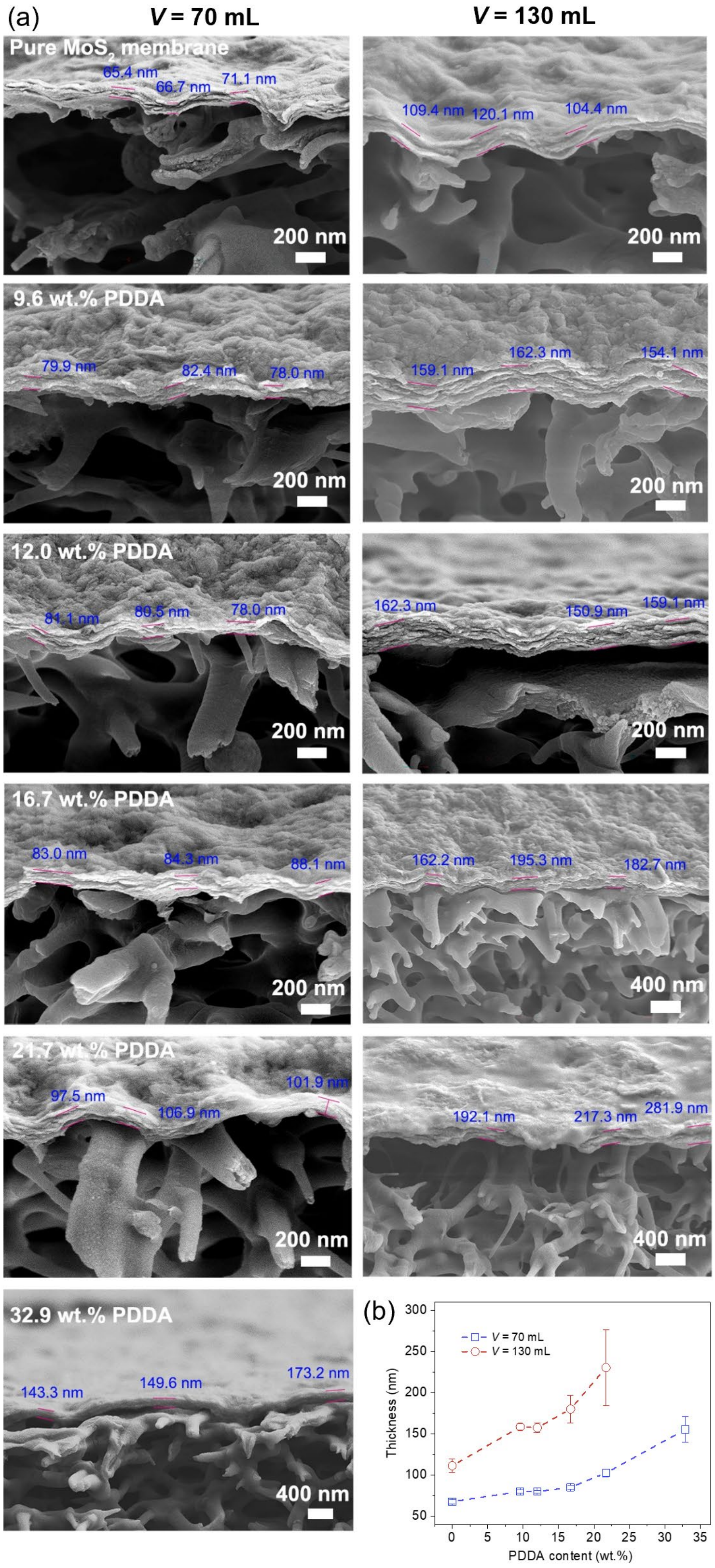

Supplementary Figure 7. (a) Cross-sectional morphologies of MoS_2-PDDA membranes prepared with different filtered dispersion volumes and varying PDDA contents. (b) Relationship between PDDA content and thickness of MoS_2-PDDA membranes. The error bars represent the standard deviations of thickness measured from three independent SEM image analyses.

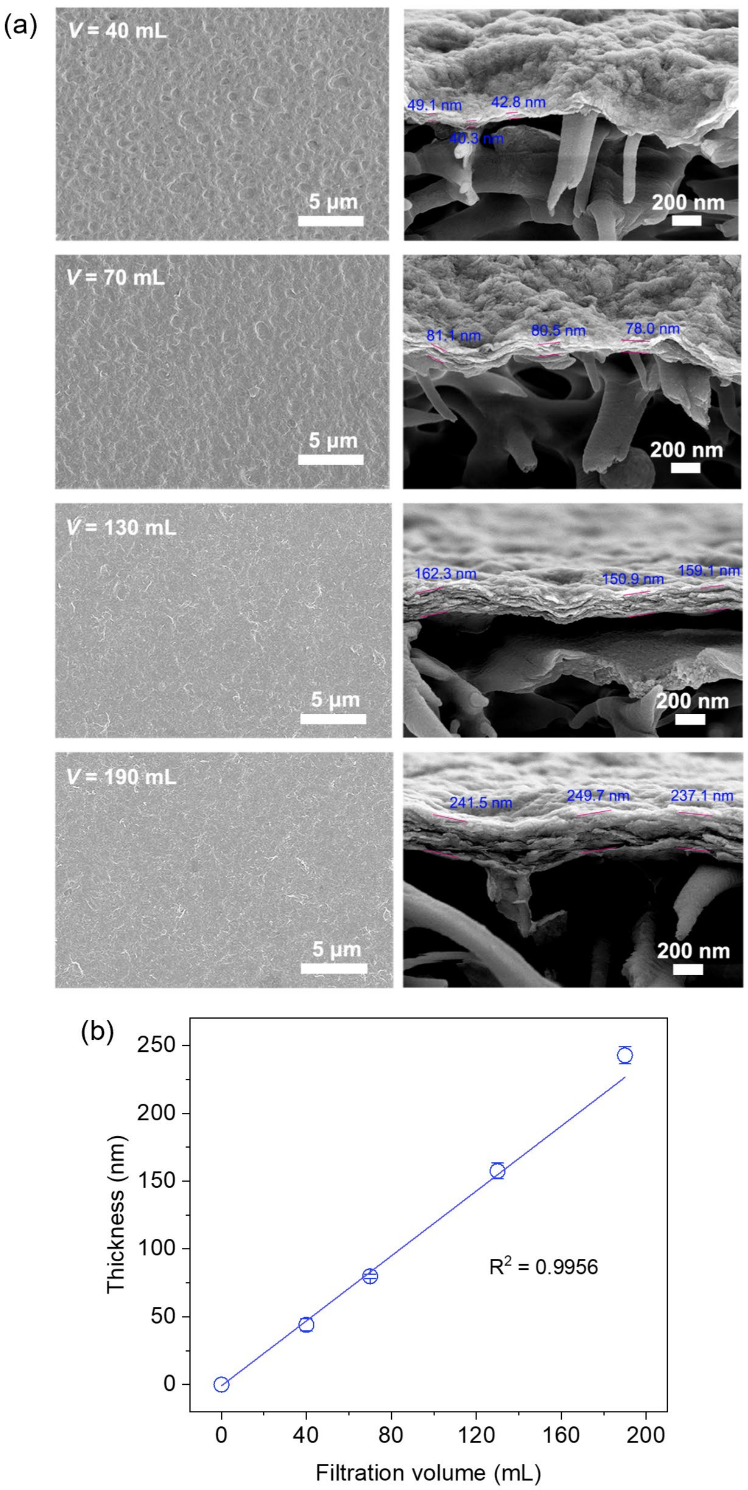

Supplementary Figure 8. (a) Surface (left) and cross-sectional (right) morphologies of MoS_2-PDDA membranes prepared with different filtration volumes (PDDA content: 12.0 wt.%). (b) Relationship between the filtration volume and membrane thickness. The error bars represent the standard deviations of thickness measured from three independent SEM image analyses.

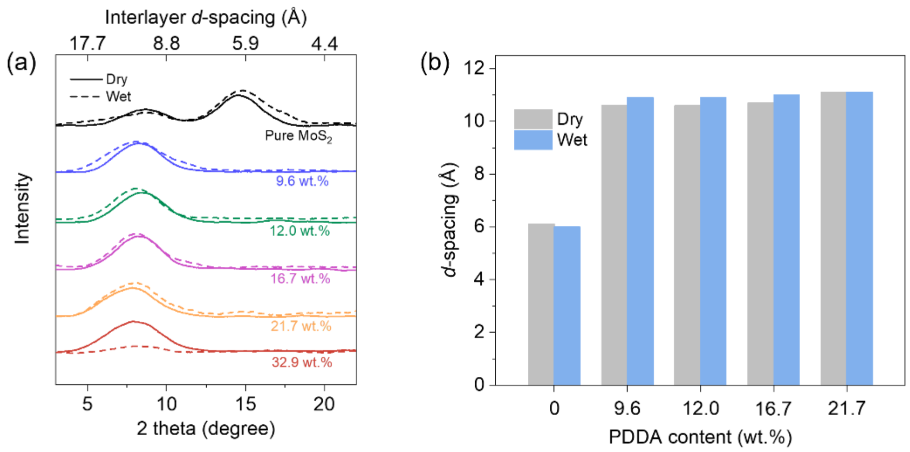

Supplementary Figure 9. XRD patterns (left) and calculated interlayer *d*-spacings (right) of MoS_2-PDDA membranes with different PDDA content in both dry and wet conditions.

It is found that MoS_2-PDDA membranes with appropriate PDDA content (9.6–21.7 wt.%) show very little difference in the obtained XRD patterns, corresponding to similar *d*-spacings. However, the membrane with an excess PDDA content (32.9 wt.%) shows a disappeared (002) peak in the wet state, suggesting the loss of the regularly stacked structure due to the swelling.

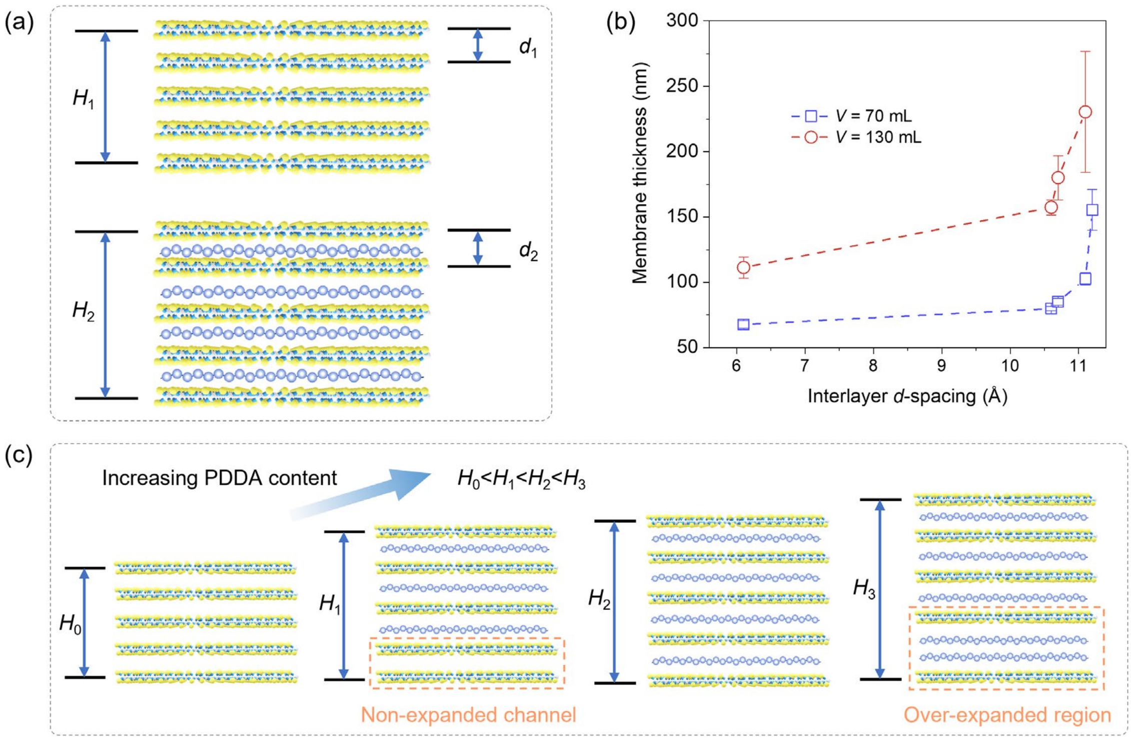

Supplementary Figure 10. (a) Schematic illustration of the linear relationship between membrane thickness and interlayer d-spacings in a perfectly stacked MoS_2-PDDA membrane. (b) The observed relationship between membrane thickness and interlayer d-spacings for our MoS_2-PDDA membranes. The error bars represent the standard deviations of thickness measured from three independent SEM image analyses. (c) Schematic illustration of the existence of non-expanded and over-expanded channels in MoS_2-PDDA membranes.

Assuming that MoS_2 nanosheets are stacked in a perfect manner with evenly distributed PDDA in between paralleled nanosheets, the increase of the membrane thickness should be linearly proportional to the expansion of d-spacings ($H_1/H_2= d_1/d_2$) (Supplementary Figure 10a). However, in our observations, the relationship between membrane thickness and d-spacings deviates significantly from the proportionality (Supplementary Figure 10b). It can be interpreted by the existence of a small percentage of non-expanded and over-expanded channels in MoS_2-PDDA membranes, resulting from insufficient and excessive PDDA incorporation, respectively (Supplementary Figure 10c).

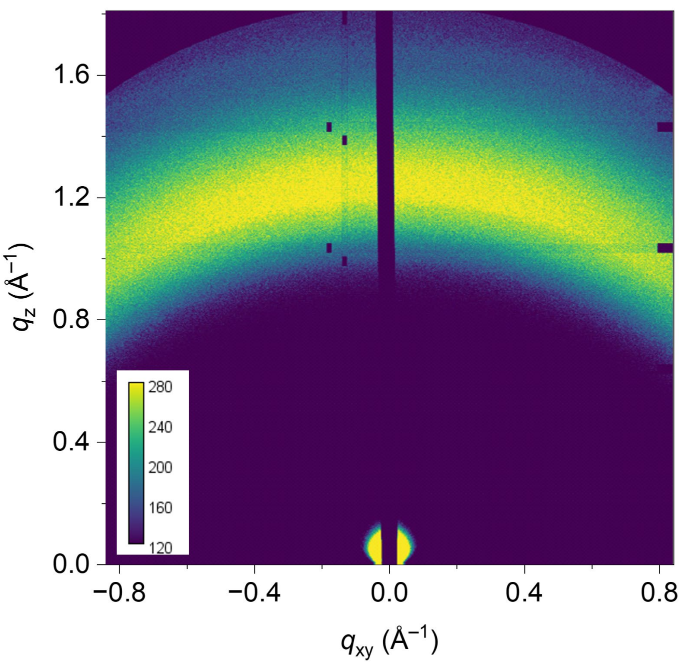

Supplementary Figure 11. The GISAXS image of the PES substrate.

The arc with stronger intensity at a higher q_z arises from the diffraction projection signal of the underlying PES substrate.

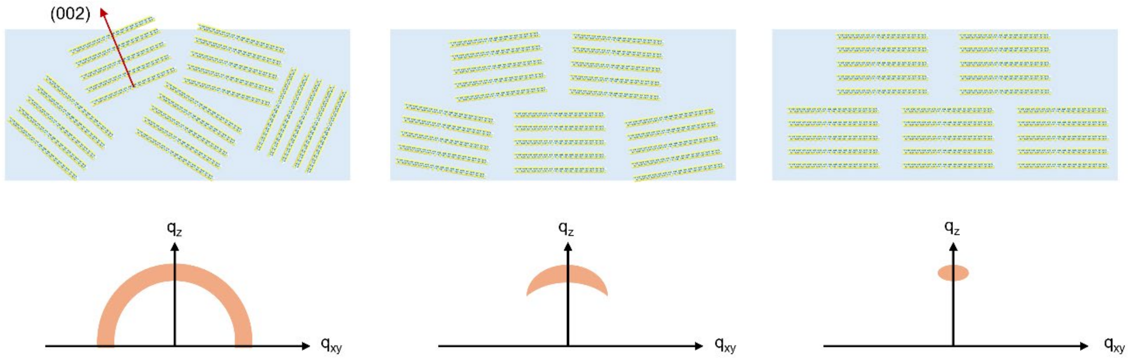

Supplementary Figure 12. Schematic illustration of the relationship between the stacking order of 2D nanosheets (top panel) and the corresponding GISAXS patterns (bottom panel).

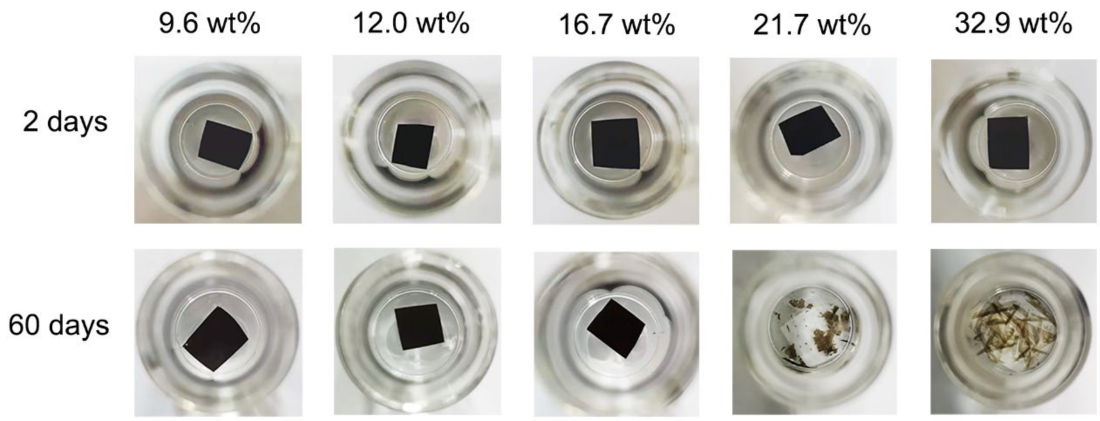

Supplementary Figure 13. Photographs of MoS_2-PDDA membranes after being immersed in water for 2 and 60 days.

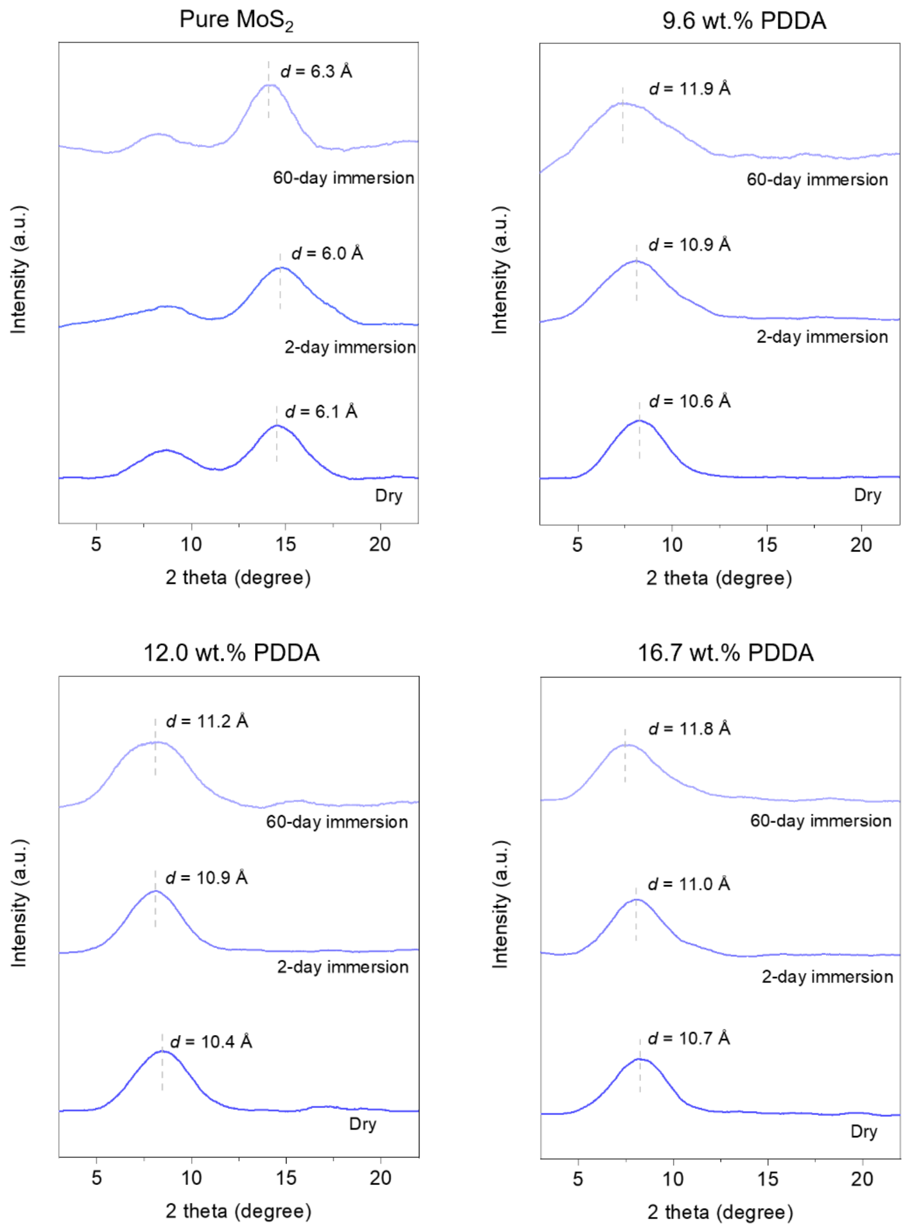

Supplementary Figure 14. XRD patterns of MoS_2-PDDA membranes with PDDA contents of 0–16.7 wt.% when immersed in water for 2 and 60 days.

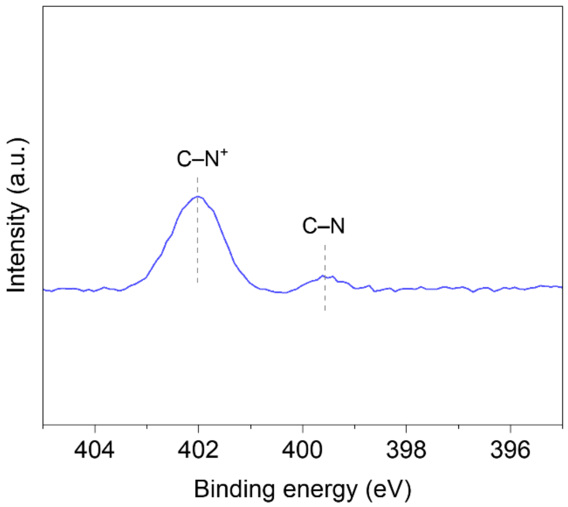

Supplementary Figure 15. High-resolution N 1*s* XPS spectrum of PDDA.

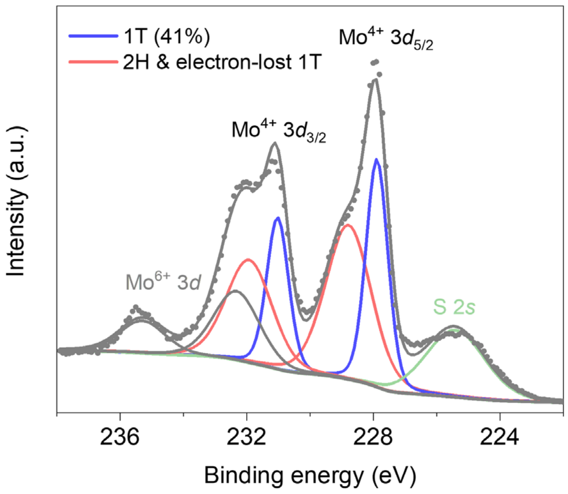

Supplementary Figure 16. High-resolution Mo 3*d* XPS spectrum of MoS_2-PDDA membranes.

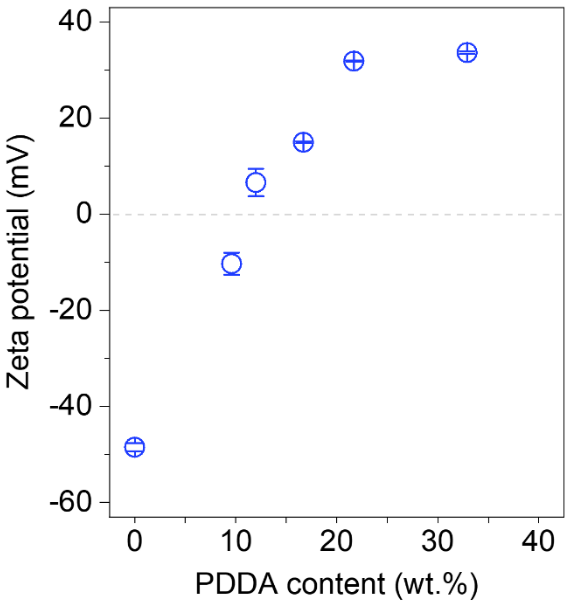

Supplementary Figure 17. Surface zeta potentials of MoS_2-PDDA membranes with different PDDA contents under neutral pH. The error bars represent the standard deviations based on three independent measurements.

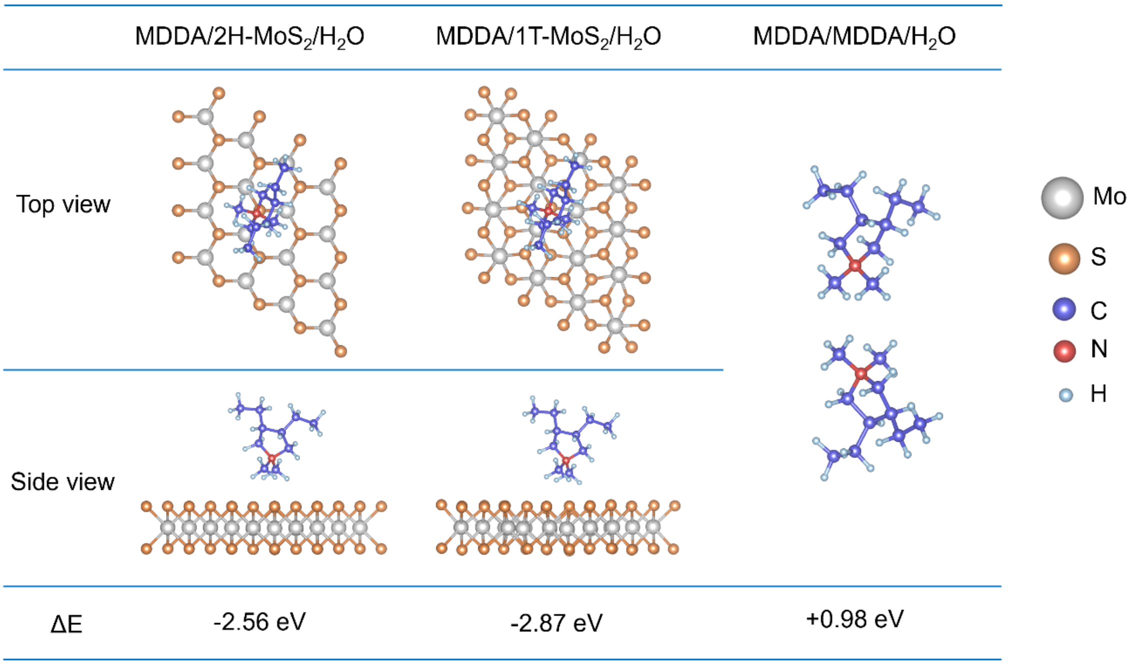

Supplementary Figure 18. Energy-optimized configurations of bonding between MDDA molecules, the MDDA molecule and the MoS_2 layer in water obtained by DFT calculations.

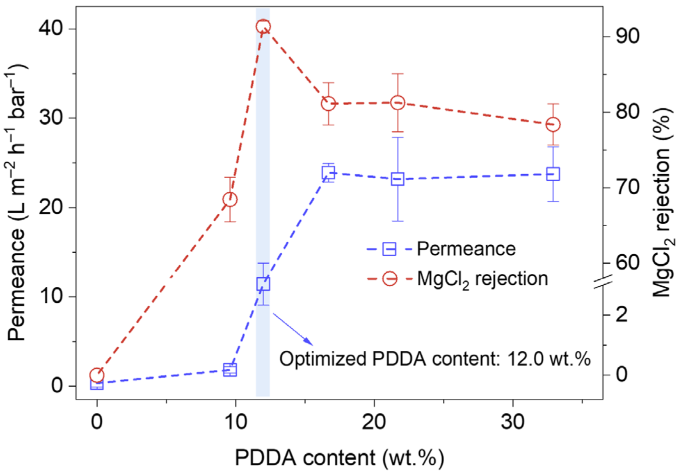

Supplementary Figure 19. Separation performance of MoS_2-PDDA membranes with different PDDA contents. The optimized PDDA content is identified as 12.0 wt.%, as indicated by the light blue region. The error bars represent the standard deviations derived from at least three independent tests.

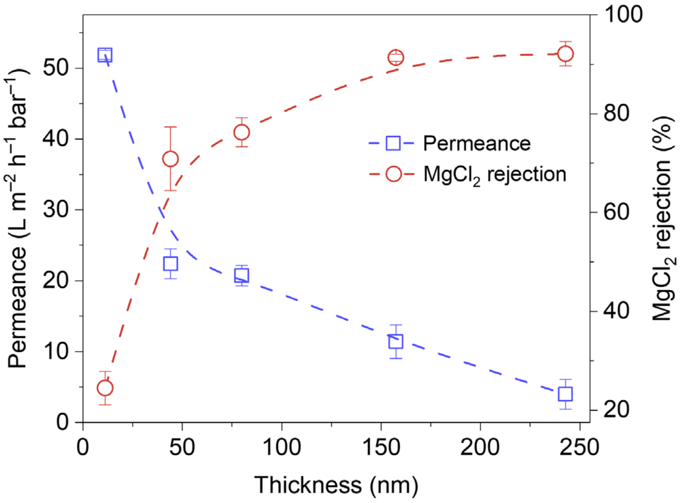

Supplementary Figure 20. Separation performance of MoS_2-PDDA membranes with different thickness. The error bars represent the standard deviations based on at least three independent tests.

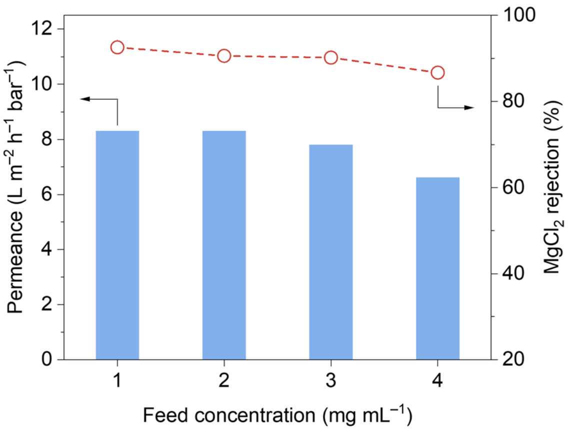

Supplementary Figure 21. Effects of feed concentration on the separation performance of MoS_2-PDDA membranes.

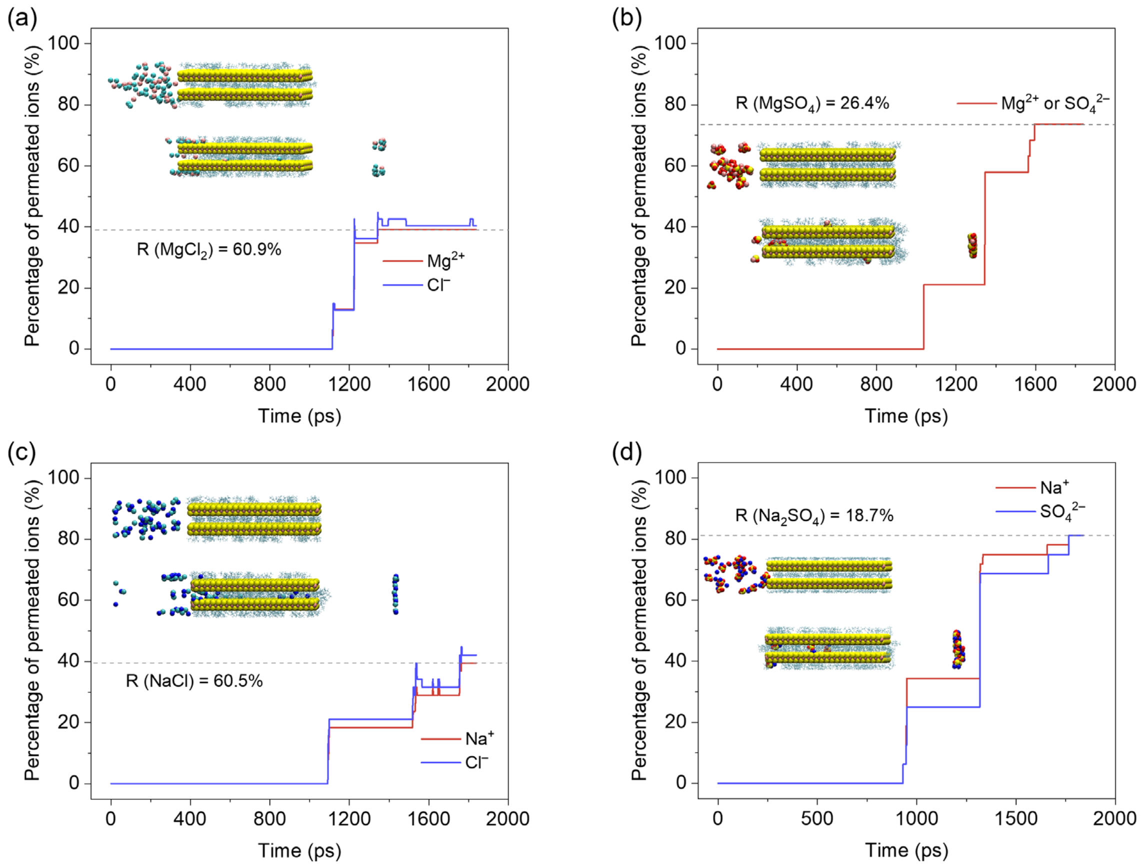

Supplementary Figure 22. Ion permeation through simulated channel of MoS_2-PDDA membranes as a function of time in the case of different salts including (a) $MgCl_2$, (b) $MgSO_4$, (c) NaCl, and (d) Na_2SO_4. The insets in each graph depict the initial (top panel) and final (bottom panel) snapshots of the simulation model.

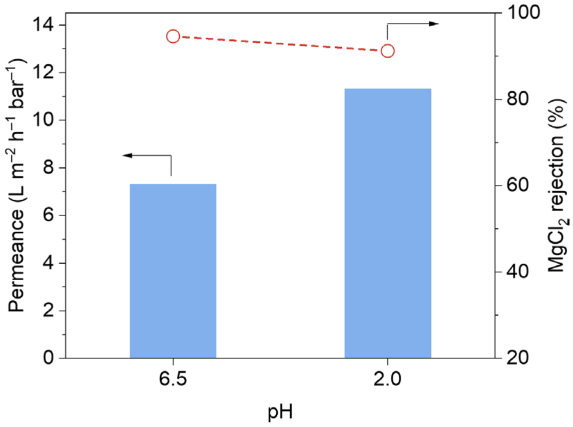

Supplementary Figure 23. Separation performance of MoS_2-PDDA membranes at different pH levels.

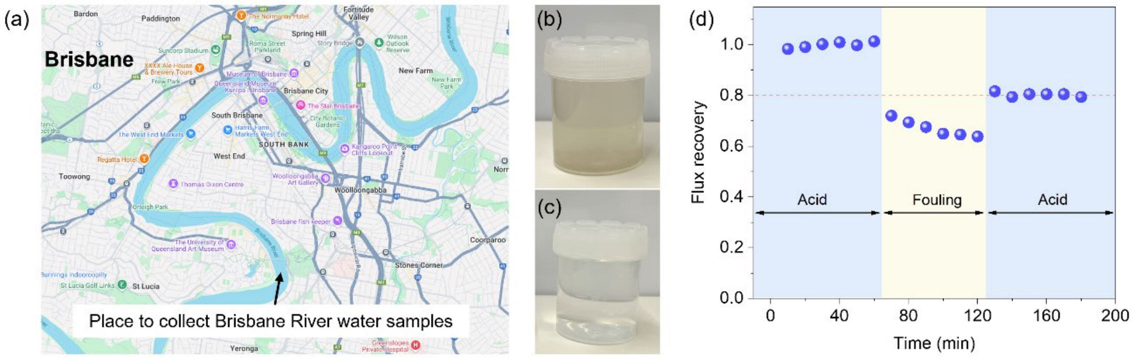

Supplementary Figure 24. (a) Location of Brisbane River (Map data ©2024 Google) for collecting water samples.

Photographs of water samples (b) before and (c) after pretreatment using microfiltration membranes. (d) Antifouling performance of MoS_2-PDDA membranes tested with pretreated Brisbane River water with an adjusted pH of 2.

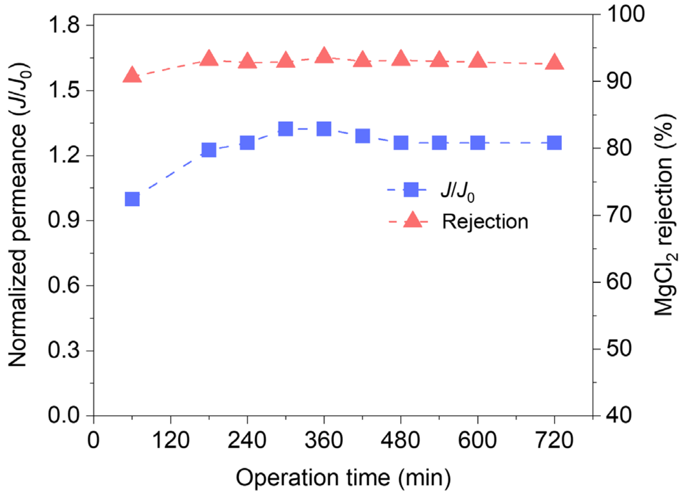

Supplementary Figure 25. Operational stability of MoS_2-PDDA membranes under the crossflow operation over a duration of 720 min. J_0 and J represent the water permeance at 60 min of operation and beyond 60 min during a total runtime of 720 min.

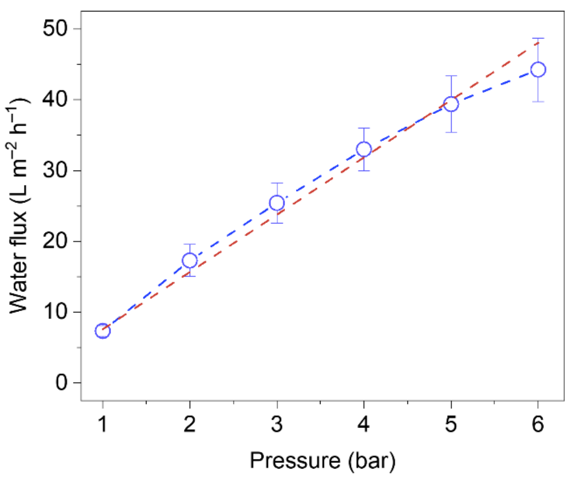

Supplementary Figure 26. Anti-pressure stability of MoS_2-PDDA membranes. The error bars represent the standard deviations based on four independent tests.

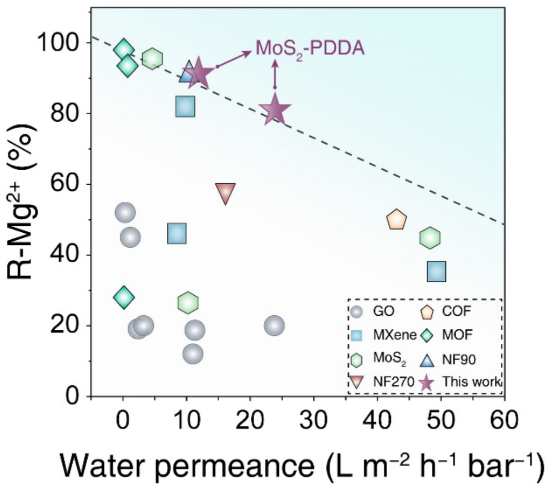

Supplementary Figure 27. Comparative analysis of the separation performance of MoS_2-PDDA membranes against other state-of-the-art nanofiltration membranes.

Supplementary Table 1. Comparison of the swelling percentage of our MoS_2-PDDA membranes with other 2D membranes reported in the literature.

2D membranes	Immersion time	Immersion medium	Swelling percentage (%)	Ref.
MoS_2-amide	24 h	Water	7	6
MoS_2-ethanol			11	
GO			86	
MXene			22	
MoS_2-EtOH	15 h	Water	~ 15	7
MoS_2-Acet			~ 7.5	
MoS_2-Met			~ –2	
GO			~ 65	
GO	6 days	Water	~ 700	8
GO-TBO	5 min	Water	~ 8	9
GO-EDA	N/A	Water	17.9	10
GO-PPD			12.1	
GO-PDA			6.7	
TU-GOF	N/A	Water	18.9	11
MPD-GOF			19.6	
MXene	N/A	Water	~ 18	12
MXene@PSS			~ 18	
MXene	3 h	Water	~ 56	13
MXene-EDTA			~ 24	
MoS_2-PDDA	2 days	Water	4.8	This work
	60 days		7.7	
	2 days	1 M H_2SO_4	8.7	
	60 days		8.7	

Supplementary Table 2. Hydrated diameters of studied ions[14, 15].

Water and ions	Bare diameter (Å)	Hydrated diameter (Å)
H_2O	2.76	2.76
Na^+	2.04	7.16
Mg^{2+}	1.44	8.56
Ni^{2+}	1.38	8.08
Co^{2+}	1.50	8.46
Cu^{2+}	1.46	8.38
Mn^{2+}	1.66	8.76
Cr^{3+}	1.24	9.22
Cl^-	3.62	6.64
SO_4^{2-}	4.60	7.58

Supplementary Table 3. Summary of the roles of intercalants in reported 2D membranes.

2D materials	Intercalant	Intercalation chemistry	"spacer"	"Regulator"	"stabilizer"	Ref.
MoS_2	Acetate or amide moieties	Covalent	✓	✓	✕	16
	Acetamide, methyl or ethyl-2-ol moieties	Covalent	✓	✓	✕	7
	Amide or ethanol moieties	Covalent	✓	✓	✕	6
	C2-, C3-, Ace-, Ben-, C3OH- or Aryl moieties	Covalent	✓	✓	✕	17
	K^+, Na^+, Li^+, or Mg^{2+}	Non-covalent	✓	✕	✕	18
	Crystal violet, sunset yellow, or neutral red	Non-covalent	✕	✓	✓	19
	Polyethyleneimine	Non-covalent	✕	✕	✓	20
Graphene oxide	K^+, Na^+, Li^+, Ca^{2+}, or Mg^{2+}	Non-covalent	✓	✕	✓	21
	Glucose	Covalent	✓	✓	✓	22
	Metal oxide nanoparticles	Non-covalent	✓	✕	✕	23
	Theanine amino acid or tannic acid	Covalent	✓	✕	✓	24
	Nanodiamonds	Non-covalent	✕	✕	✓	25
	Polyamine macromolecules	Non-covalent	✓	✓	✕	26
	Polycyclic π-conjugated cations	Non-covalent	✓	✕	✓	9
	Thiourea or m-phenylenediamine	Covalent	✓	✕	✓	11
	4-sulfocalix[4]arene	Covalent	✓	✓	✕	27
MXene	Poly(sodium 4-styrene sulfonate)	Non-covalent	✓	✓	✕	12
	Alginate hydrogel pillars	Non-covalent	✓	✕	✓	28
	Solvent molecules	Non-covalent	✓	✕	✕	29
	Al^{3+}	Non-covalent	✓	✕	✓	30
g-C_3N_4	Sulfonated molecules	Non-covalent	✓	✕	✓	31
Boron nitride	Ionic liquid	Non-covalent	✓	✓	✕	32
MoS_2	PDDA	Non-covalent	✓	✓	✓	This work

Intercalation strategies have been widely employed in the functionalization of 2D membranes, involving the incorporation of various intercalants such as ions, molecules, and polymers. Upon conducting a thorough literature review, it is noteworthy that most intercalants within 2D membranes are found to serve limited roles. These are typically categorized into one or two of the following functions: (a) As a "spacer" to define the interlayer channel size; (b) As a "regulator" to selectively accelerate ("carrier") or retard ("barrier") the transport of specific species; (c) As a "stabilizer" to maintain channel size and chemistry.

Supplementary Table 4. Comparison of the separation performance with state-of-the-art 2D membranes reported in the literature.

Membrane	Water permeance (L m^{-2} h^{-1} bar^{-1})	Feed concentration	Thickness (nm)	Rejection (%)			Ref.
				$MgCl_2$	Na_2SO_4	NaCl	
GO	0.97	0.01 M	150	-	84	71	9
GO-TBO	0.41	0.01 M	134	-	90	81	
GO	2.1	1 g L^{-1}	58	-	84.3	30.4	33
ZIF-8@f-GOm	49.8	1 g L^{-1}	105	-	52.9	30.3	
MoS_2-amide	5.2	0.03 M	500	-	85	77	6
MoS_2-ethanol	6.6	0.03 M	500	-	79	72	
GO/MoS_2	10.2	0.001 M	> 1000	26.5	65.2	43.2	34
G-CNTm	11.3	0.01 M	40	18.7	83.5	51.4	35
GO-PDDA	15.8	0.05 g L^{-1}	130	95.2	-	-	36
GO-PEI	11.4		130	93.1	-	-	
GO-PAH	13.2		110	77.8	-	-	
GO/TiO2-PDDA	51.2		300	93.2	-	-	
MoS_2-Acet	9.3	0.1 M	500	-	-	78.5	7
MoS_2-EtOH	13.4	0.1 M	500	-	-	88.2	
MoS_2-Met	15.9	0.1 M	500	-	-	95	
BPPO/EDA/GO	4.1	1 g L^{-1}	~200	-	56.2	36.3	37
mul-EGO-60	23.8	-	52	~20	78.3	~20	38
MoS_2/PEI	4.6	0.01 M	1800	95.5	~30	~60	20
GO	11	2 g L^{-1}	150	~12	~65	~24	39
GO	2.4	0.003 M	50	19.1	79.5	45.2	40
CCG	0.36	0.5 g L^{-1}	< 100	~52	~98	~90	41
TMPyP/GO	1.16	2 g L^{-1}	-	45	87.7	~30	42
uGNMs	3.26	0.02 M	53	~20	~60	~40	43
Aceta-MoS_2	1.5	0.017 M Na_2SO_4	-	-	92	~55	16
Amide-MoS_2	0.7	0.034 M NaCl		-	89.0	-	
MoS_2/GO	48.27	1 g L^{-1}	-	44.85	66.76	56.02	44
Ti_3C_2Tx-Mn-SAT	16.5	0.05 g L^{-1}	50	-	84	-	28
Mxene	49.3	0.05 g L^{-1}	50	35.3	-	-	45
SC-Mxene	9.8			82	~40	~50	
MXene-derived	8.5	0.01 M	-	46.1	75.9	55.3	46
COF@CNFs	~43	1 g L^{-1}	-	~50	96.8	~25	47
pDA/TpPa(W/E)-COF	~55	1 g L^{-1}	125	70.3	99.5	49.2	24
UiO-66	0.14	2 g L^{-1}	~ 2000	98.0	~45	-	48
MOF-303	0.74	1 g L^{-1}	~ 4000	93.5	96.0	33.2	49
UiO-66(Zr)-$(OH)_2$	0.16	2 g L^{-1}	~ 3500	28	-	26	50
MoS_2-PDDA (12.0 wt.%)	11.4	1 g L^{-1}	157.4	91.4	11.2	41.8	This work
MoS_2-PDDA (16.7 wt.%)	23.9		180.1	81.1	-	-	

Supplementary Table 5. Comparison of the separation performance and acid stability with previously reported acid-tolerant membranes.

Membrane	Water permeance ($L\ m^{-2}\ h^{-1}\ bar^{-1}$)	$MgCl_2$ rejection (%)	Acid stability	pH	Ref.
Polysulfonamide	6.8	92.4	2 M H_2SO_4, 30 days	–0.6	51
SPEEK/polysulfonamide	1.7	78.0	0.8 M H_2SO_4, 1 day	−0.2	52
PEI/CC	3.0	90.0	0.1 M HNO_3, 35 days	1.0	53
PEI-CC	2.5	94.8	0.8 M HCl, 4 days	0.1	54
COF/polyamide	8.5	72.0	0.01 M HCl, 5 days	2.0	55
Crosslinked PEI	0.7	90.0	0.02 M HCl, 30 days	1.7	56
PEI/BCMP	10.1	95.6	0.03 M HCl, 1 day	1.5	57
PVA-SMPTES	2.2	47.8	1.5 M H_2SO_4, 30 days	–0.5	58
Poly(aryl cyanurate)	1.8	32.1	0.1 M HNO_3, 66 days	1.0	59
Poly(quaternary ammonium)	2.9	97.0	1.5 M H_2SO_4, 28 days	–0.5	60
NP030	1.8	24.0	1.5 M H_2SO_4, 28 days	–0.5	60
MoS_2-PDDA	11.4	91.4	1 M H_2SO_4, 12 days	–0.3	Our study

Supplementary References

1. Lindahl E, Hess B, van der Spoel D. GROMACS 3.0: a package for molecular simulation and trajectory analysis. *Molecular modeling annual* 2001, **7**(8)**:** 306-317.

2. Garberoglio G. OBGMX: A web-based generator of GROMACS topologies for molecular and periodic systems using the universal force field. *J. Comput. Chem.* 2012, **33**(27)**:** 2204-2208.

3. Hess B, Bekker H, Berendsen HJC, Fraaije JGEM. LINCS: A linear constraint solver for molecular simulations. *J. Comput. Chem.* 1997, **18**(12)**:** 1463-1472.

4. Feller SE, Zhang Y, Pastor RW, Brooks BR. Constant pressure molecular dynamics simulation: The Langevin piston method. *J. Chem. Phys.* 1995, **103**(11)**:** 4613-4621.

5. Humphrey W, Dalke A, Schulten K. VMD: Visual molecular dynamics. *J. Mol. Graphics* 1996, **14**(1)**:** 33-38.

6. Mei L, Cao Z, Ying T, Yang R, Peng H, Wang G, *et al.* Simultaneous Electrochemical Exfoliation and Covalent Functionalization of MoS2 Membrane for Ion Sieving. *Adv. Mater.* 2022, **34:** 2201416.

7. Ries L, Petit E, Michel T, Diogo CC, Gervais C, Salameh C, *et al.* Enhanced sieving from exfoliated MoS2 membranes via covalent functionalization. *Nat. Mater.* 2019, **18**(10)**:** 1112-1117.

8. Zheng S, Tu Q, Urban JJ, Li S, Mi B. Swelling of Graphene Oxide Membranes in Aqueous Solution: Characterization of Interlayer Spacing and Insight into Water Transport Mechanisms. *ACS Nano* 2017, **11**(6)**:** 6440-6450.

9. Wang Z, Ma C, Xu C, Sinquefield SA, Shofner ML, Nair S. Graphene oxide nanofiltration membranes for desalination under realistic conditions. *Nat. Sustain.* 2021, **4**(5)**:** 402-408.

10. Zhang M, Mao Y, Liu G, Liu G, Fan Y, Jin W. Molecular Bridges Stabilize Graphene Oxide Membranes in Water. *Angew. Chem. Int. Ed.* 2020, **59**(4)**:** 1689-1695.

11. Yuan B, Wang M, Wang B, Yang F, Quan X, Tang CY, *et al.* Cross-linked Graphene Oxide Framework Membranes with Robust Nano-Channels for Enhanced Sieving Ability. *Environ. Sci. Technol.* 2020, **54**(23)**:** 15442-15453.

12. Lu Z, Wu Y, Ding L, Wei Y, Wang H. A Lamellar MXene (Ti3C2Tx)/PSS Composite Membrane for Fast and Selective Lithium-Ion Separation. *Angew. Chem. Int. Ed.* 2021, **60**(41)**:** 22265-22269.

13. Xu R, Kang Y, Zhang W, Pan B, Zhang X. Two-dimensional MXene membranes with biomimetic sub-nanochannels for enhanced cation sieving. *Nat. Commun.* 2023, **14**(1)**:** 4907.

14. Marcus Y. Ionic radii in aqueous solutions. *Chem. Rev.* 1988, **88**(8)**:** 1475-1498.

15. Nightingale ER, Jr. Phenomenological Theory of Ion Solvation. Effective Radii of Hydrated Ions. *J. Phys. Chem. A* 1959, **63**(9)**:** 1381-1387.

16. Hoenig E, Strong SE, Wang M, Radhakrishnan JM, Zaluzec NJ, Skinner JL*, et al.* Controlling the Structure of MoS2 Membranes via Covalent Functionalization with Molecular Spacers. *Nano Lett.* 2020, **20**(11)**:** 7844-7851.

17. Wang W, Onofrio N, Petit E, Karamoko BA, Wu H, Liu J*, et al.* High-surface-area functionalized nanolaminated membranes for energy-efficient nanofiltration and desalination in forward osmosis. *Nat. Water* 2023, **1**(2)**:** 187-197.

18. Chu C, Fu C-F, Zhang P, Pan T, Ai X, Wu Y*, et al.* Precise ångström controlling the interlayer channel of MoS2 membranes by cation intercalation. *J. Membr. Sci.* 2020, **615:** 118520.

19. Hirunpinyopas W, Prestat E, Worrall SD, Haigh SJ, Dryfe RAW, Bissett MA. Desalination and Nanofiltration through Functionalized Laminar MoS2 Membranes. *ACS Nano* 2017, **11**(11)**:** 11082-11090.

20. Zhang H, Taymazov D, Li M-P, Huang Z-H, Liu W-L, Zhang X*, et al.* Construction of MoS2 composite membranes on ceramic hollow fibers for efficient water desalination. *J. Membr. Sci.* 2019, **592:** 117369.

21. Chen L, Shi G, Shen J, Peng B, Zhang B, Wang Y*, et al.* Ion sieving in graphene oxide membranes via cationic control of interlayer spacing. *Nature* 2017, **550**(7676)**:** 380-383.

22. Guan K, Guo Y, Li Z, Jia Y, Shen Q, Nakagawa K*, et al.* Deformation constraints of graphene oxide nanochannels under reverse osmosis. *Nat. Commun.* 2023, **14**(1)**:** 1016.

23. Zhang W, Xu H, Xie F, Ma X, Niu B, Chen M*, et al.* General synthesis of ultrafine metal oxide/reduced graphene oxide nanocomposites for ultrahigh-flux nanofiltration membrane. *Nat. Commun.* 2022, **13**(1)**:** 471.

24. Thebo KH, Qian X, Zhang Q, Chen L, Cheng H-M, Ren W. Highly stable graphene-oxide-based membranes with superior permeability. *Nat. Commun.* 2018, **9**(1)**:** 1486.

25. Huang G, Ghalei B, Pournaghshband Isfahani A, Karahan HE, Terada D, Qin D*, et al.* Overcoming humidity-induced swelling of graphene oxide-based hydrogen membranes using charge-compensating nanodiamonds. *Nat. Energy* 2021, **6**(12)**:** 1176-1187.

26. Andreeva DV, Trushin M, Nikitina A, Costa MCF, Cherepanov PV, Holwill M*, et al.* Two-dimensional adaptive membranes with programmable water and ionic channels. *Nat.*

Nanotechnol. 2021, **16**(2)**:** 174-180.

27. Yu W, Wei C, Zhang K, Zhang J, Ge Z, Liang X*, et al.* Host–Guest Recognition Boosts Biomimetic Mono/Multivalent Cation Separation. *Environ. Sci. Technol.* 2023, **57**(14)**:** 5861-5871.

28. Wang J, Zhang Z, Zhu J, Tian M, Zheng S, Wang F*, et al.* Ion sieving by a two-dimensional Ti3C2Tx alginate lamellar membrane with stable interlayer spacing. *Nat. Commun.* 2020, **11**(1)**:** 3540.

29. Kang Y, Hu T, Wang Y, He K, Wang Z, Hora Y*, et al.* Nanoconfinement enabled non-covalently decorated MXene membranes for ion-sieving. *Nat. Commun.* 2023, **14**(1)**:** 4075.

30. Ding L, Li L, Liu Y, Wu Y, Lu Z, Deng J*, et al.* Effective ion sieving with Ti3C2Tx MXene membranes for production of drinking water from seawater. *Nat. Sustain.* 2020, **3**(4)**:** 296-302.

31. Ran J, Pan T, Wu Y, Chu C, Cui P, Zhang P*, et al.* Endowing g-C3N4 Membranes with Superior Permeability and Stability by Using Acid Spacers. *Angew. Chem. Int. Ed.* 2019, **58**(46)**:** 16463-16468.

32. Dou H, Jiang B, Xu M, Zhang Z, Wen G, Peng F*, et al.* Boron Nitride Membranes with a Distinct Nanoconfinement Effect for Efficient Ethylene/Ethane Separation. *Angew. Chem. Int. Ed.* 2019, **58**(39)**:** 13969-13975.

33. Zhang W-H, Yin M-J, Zhao Q, Jin C-G, Wang N, Ji S*, et al.* Graphene oxide membranes with stable porous structure for ultrafast water transport. *Nat. Nanotechnol.* 2021, **16**(3)**:** 337-343.

34. Zhang P, Gong J-L, Zeng G-M, Song B, Cao W, Liu H-Y*, et al.* Novel "loose" GO/MoS2 composites membranes with enhanced permeability for effective salts and dyes rejection at low pressure. *J. Membr. Sci.* 2019, **574:** 112-123.

35. Han Y, Jiang Y, Gao C. High-Flux Graphene Oxide Nanofiltration Membrane Intercalated by Carbon Nanotubes. *ACS Appl. Mater. Interfaces* 2015, **7**(15)**:** 8147-8155.

36. Zhang M, Guan K, Ji Y, Liu G, Jin W, Xu N. Controllable ion transport by surface-charged graphene oxide membrane. *Nat. Commun.* 2019, **10**(1)**:** 1253.

37. Meng N, Zhao W, Shamsaei E, Wang G, Zeng X, Lin X*, et al.* A low-pressure GO nanofiltration membrane crosslinked via ethylenediamine. *J. Membr. Sci.* 2018, **548:** 363-371.

38. Wang Q, Zhao G, Li C, Meng H. Orderly stacked ultrathin graphene oxide membranes on a macroporous tubular ceramic substrate. *J. Membr. Sci.* 2019, **586:** 177-184.

39. Wei Y, Zhang Y, Gao X, Yuan Y, Su B, Gao C. Declining flux and narrowing nanochannels

under wrinkles of compacted graphene oxide nanofiltration membranes. *Carbon* 2016, **108:** 568-575.

40. Mo Y, Zhao X, Shen Y-x. Cation-dependent structural instability of graphene oxide membranes and its effect on membrane separation performance. *Desalination* 2016, **399:** 40-46.

41. Guan K, Jia Y, Lin Y, Wang S, Matsuyama H. Chemically Converted Graphene Nanosheets for the Construction of Ion-Exclusion Nanochannel Membranes. *Nano Lett.* 2021, **21**(8)**:** 3495-3502.

42. Xu X-L, Lin F-W, Du Y, Zhang X, Wu J, Xu Z-K. Graphene Oxide Nanofiltration Membranes Stabilized by Cationic Porphyrin for High Salt Rejection. *ACS Appl. Mater. Interfaces* 2016, **8**(20)**:** 12588-12593.

43. Han Y, Xu Z, Gao C. Ultrathin Graphene Nanofiltration Membrane for Water Purification. *Adv. Funct. Mater.* 2013, **23**(29)**:** 3693-3700.

44. Ma J, Tang X, He Y, Fan Y, Chen J, HaoYu. Robust stable MoS2/GO filtration membrane for effective removal of dyes and salts from water with enhanced permeability. *Desalination* 2020, **480:** 114328.

45. Meng B, Liu G, Mao Y, Liang F, Liu G, Jin W. Fabrication of surface-charged MXene membrane and its application for water desalination. *J. Membr. Sci.* 2021, **623:** 119076.

46. Sun Y, Li S, Zhuang Y, Liu G, Xing W, Jing W. Adjustable interlayer spacing of ultrathin MXene-derived membranes for ion rejection. *J. Membr. Sci.* 2019, **591:** 117350.

47. Yang H, Yang L, Wang H, Xu Z, Zhao Y, Luo Y*, et al.* Covalent organic framework membranes through a mixed-dimensional assembly for molecular separations. *Nat. Commun.* 2019, **10**(1)**:** 2101.

48. Liu X, Demir NK, Wu Z, Li K. Highly Water-Stable Zirconium Metal–Organic Framework UiO-66 Membranes Supported on Alumina Hollow Fibers for Desalination. *J. Am. Chem. Soc.* 2015, **137**(22)**:** 6999-7002.

49. Cong S, Yuan Y, Wang J, Wang Z, Kapteijn F, Liu X. Highly Water-Permeable Metal–Organic Framework MOF-303 Membranes for Desalination. *J. Am. Chem. Soc.* 2021, **143**(48)**:** 20055-20058.

50. Wang X, Zhai L, Wang Y, Li R, Gu X, Yuan YD*, et al.* Improving Water-Treatment Performance of Zirconium Metal-Organic Framework Membranes by Postsynthetic Defect Healing. *ACS Appl. Mater. Interfaces* 2017, **9**(43)**:** 37848-37855.

51. Wang H, Wei Z, Wang H, Jiang H, Li Y, Wu C. An acid-stable positively charged

polysulfonamide nanofiltration membrane prepared by interfacial polymerization of polyallylamine and 1,3-benzenedisulfonyl chloride for water treatment. *RSC Adv.* 2019, **9**(4)**:** 2042-2054.

52. Zhu Y, Dou P, He H, Lan H, Xu S, Zhang Y*, et al.* Improvement of permeability and rejection of an acid resistant polysulfonamide thin-film composite nanofiltration membrane by a sulfonated poly(ether ether ketone) interlayer. *Sep. Purif. Technol.* 2020, **239:** 116528.

53. Lee KP, Zheng J, Bargeman G, Kemperman AJB, Benes NE. pH stable thin film composite polyamine nanofiltration membranes by interfacial polymerisation. *J. Membr. Sci.* 2015, **478:** 75-84.

54. Yu L, Zhang Y, Xu L, Liu Q, Borjigin B, Hou D*, et al.* One step prepared Janus acid-resistant nanofiltration membranes with opposite surface charges for acidic wastewater treatment. *Sep. Purif. Technol.* 2020, **250:** 117245.

55. Jiang Y, Li S, Su J, Lv X, Liu S, Su B. Two dimensional COFs as ultra-thin interlayer to build TFN hollow fiber nanofiltration membrane for desalination and heavy metal wastewater treatment. *J. Membr. Sci.* 2021, **635:** 119523.

56. Yun T, Kwak S-Y. Recovery of hydrochloric acid using positively-charged nanofiltration membrane with selective acid permeability and acid resistance. *J. Environ. Manage.* 2020, **260:** 110001.

57. Gu K, Pang S, Yang B, Ji Y, Zhou Y, Gao C. Polyethyleneimine/4,4′-Bis(chloromethyl)-1,1′-biphenyl nanofiltration membrane for metal ions removal in acid wastewater. *J. Membr. Sci.* 2020, **614:** 118497.

58. Zhang Y, Guo M, Pan G, Yan H, Xu J, Shi Y*, et al.* Preparation and properties of novel pH-stable TFC membrane based on organic–inorganic hybrid composite materials for nanofiltration. *J. Membr. Sci.* 2015, **476:** 500-507.

59. Elshof MG, de Vos WM, de Grooth J, Benes NE. On the long-term pH stability of polyelectrolyte multilayer nanofiltration membranes. *J. Membr. Sci.* 2020, **615:** 118532.

60. Jeon S, Kim H, Choi J, Kim JF, Park HB, Lee J-H. Extreme pH-Resistant, Highly Cation-Selective Poly(Quaternary Ammonium) Membranes Fabricated via Menshutkin Reaction-Based Interfacial Polymerization. *Adv. Funct. Mater.* 2023, **33**(22)**:** 2300183.
