# Supplementary material for: Multifunctional intercalants create stable subnanochannels in MoS2 membranes for wastewater treatment
Source: Nat Commun. 2025 Sep 24;16:8353. doi: 10.1038/s41467-025-58409-x (PMC12460844; doi:10.1038/s41467-025-58409-x)
Supplement: Supplementary file 2 — Transparent Peer Review file [file 41467_2025_58409_MOESM2_ESM.pdf]

nature portfolio

Peer Review File

Multifunctional intercalants create stable subnanochannels in MoS2 membranes for wastewater treatment

Corresponding Author: Professor Xiwang Zhang

This manuscript has been previously reviewed at another journal. This document only contains information relating to versions considered at Nature Communications.

This file contains all reviewer reports in order by version, followed by all author rebuttals in order by version.

Version 0:

Reviewer comments:

Reviewer #1

(Remarks to the Author)
The authors have addressed some comments in the revised manuscript. Several major issues remain unresolved:
1. In this version, the authors attempted to highlight the advantage of their membranes in highly acidic conditions. However, the mechanisms behind their acid resistance are unclear. Is it due to enhanced covalent bonding (which may improve resistance to hydrolysis) or the Van der Waals forces between adjacent nanosheets?
2. The authors claim that in highly acidic conditions, negatively charged foulants become protonated at low pH, reducing their negative charge and consequently their interaction with the membrane surface, thereby mitigating fouling. However, under such conditions, the protonated molecules are neutrally charged, making electrostatic interactions negligible. In this scenario, any type of membrane—whether negatively or positively charged—would be less prone to fouling. This calls into question of their membrane's advantage of fouling resistance.
3. Another major issue is the use of real river water to demonstrate the membrane's fouling propensity. However, such river water does not reflect the highly acidic conditions claimed in the paper. This inconsistency undermines the relevance of the results to the acidic environment emphasized in the study.
4. Based on the current analysis (XPS and DFT), the evidence supporting electron transfer between the C–N+ group and the 1T-MoS2 phase remains unconvincing. This is particularly questionable given the potential transition of MoS2 nanosheets under ambient conditions.

Reviewer #3

(Remarks to the Author)
The manuscript has been reviewed previously. All my concerns have been well-solved and the paper has revised a lot. Thus, it has been in a good shape, which can be published now.

Version 1:

Reviewer comments:

Reviewer #1

(Remarks to the Author)
The responses and revisions are fine.

Open Access This Peer Review File is licensed under a Creative Commons Attribution 4.0 International License, which permits use, sharing, adaptation, distribution and reproduction in any medium or format, as long as you give appropriate credit to the original author(s) and the source, provide a link to the Creative Commons license, and indicate if changes were made.
In cases where reviewers are anonymous, credit should be given to 'Anonymous Referee' and the source.
The images or other third party material in this Peer Review File are included in the article's Creative Commons license, unless indicated otherwise in a credit line to the material. If material is not included in the article's Creative Commons license and your intended use is not permitted by statutory regulation or exceeds the permitted use, you will need to obtain permission directly from the copyright holder.
To view a copy of this license, visit https://creativecommons.org/licenses/by/4.0/

Reviewer #1 (Comments for the Author)

Q1. *In this version, the authors attempted to highlight the advantage of their membranes in highly acidic conditions. However, the mechanisms behind their acid resistance are unclear. Is it due to enhanced covalent bonding (which may improve resistance to hydrolysis) or the Van der Waals forces between adjacent nanosheets?*

Response: We appreciate the reviewer's insightful comments. The stability of our MoS_2-PDDA membranes in acidic environments (i.e., acid resistance) can be understood by the interlocking effect enabled by the **persistent interaction between QAs and the 1T-MoS_2** under nanoconfinement.

It has been reported that protons tend to interact with 1T-MoS_2 to form covalent S–H bonds through electron transfer, leading to channel shrinkage and microstructural instability in pristine MoS_2 membranes (*Nature* 2023, 616, 719–723) (see **Introduction** section in the manuscript). In the presence of PDDA, which also shows a strong tendency to interact with 1T-MoS_2, there is likely competition between protons and QAs for interaction with 1T-MoS_2. We experimentally demonstrate that **QAs preferentially interact with 1T-MoS_2 over protons**, as evidenced by the **slight decrease in the percentage of neutralized C–N** from 53.8 to 46.5% (**Fig. 3b and c**). In other words, the favourable interaction between QAs and the 1T-MoS_2, enabled by electron density shifts, remains intact even in acidic environments. **As long as QAs remain anchored to the MoS_2 channel walls, the interlocking effect imposed by the long-chain PDDA will prevent the channel from either shrinking or expanding, thereby ensuring the structural integrity and satisfactory stability of the membrane in acidic environments.**

Q2. *The authors claim that in highly acidic conditions, negatively charged foulants become protonated at low pH, reducing their negative charge and consequently their interaction with the membrane surface, thereby mitigating fouling. However, under such conditions, the protonated molecules are neutrally charged, making electrostatic interactions negligible. In this scenario, any type of membrane—whether negatively or positively charged—would be less prone to fouling. This calls into question of their membrane's advantage of fouling resistance.*

Response: We appreciate the reviewer's comments. We acknowledge that **membranes in acidic environments are inherently less prone to fouling** due to significantly weakened electrostatic interactions. For this reason, we focused our study on evaluating the separation performance and stability of our MoS_2-PDDA membranes, which are more critical for their

practical application in such conditions, rather than on fouling resistance. More importantly, from the perspective of the functionalization–structure–performance relationship in our membranes, **fouling resistance is not closely connected with the elucidated roles of the intercalants**, further supporting our decision to prioritize the core objectives of the study.

Q3. *Another major issue is the use of real river water to demonstrate the membrane's fouling propensity. However, such river water does not reflect the highly acidic conditions claimed in the paper. This inconsistency undermines the relevance of the results to the acidic environment emphasized in the study.*

Response: As stated in our manuscript, **we adjusted the pH of the river water to 2** to simulate an acidic environment.

Q4. *Based on the current analysis (XPS and DFT), the evidence supporting electron transfer between the C–N⁺ group and the 1T-MoS_2 phase remains unconvincing. This is particularly questionable given the potential transition of MoS_2 nanosheets under ambient conditions.*

Response: We appreciate the reviewer's comments. It is widely acknowledged that **high-resolution XPS represents a powerful tool for unveiling the electron density shift** in materials science. Herein, our high-resolution N 1s XPS spectra show **partial neutralization of the $C–N^+$** in PDDA within subnanometric MoS_2 channels, confirming **electron density shifts** at the QA/1T-MoS_2 interface (**Fig. 3a and b**). Moreover, DFT calculations, especially the Bader charge analysis, serve as further validation of such electron density shifts. As such, we are confident that our analysis affords convincing evidence supporting the electron density shifts.

We acknowledge that a significant phase transition of MoS_2 nanosheets from 1T to 2H could potentially undermine the validation of electron density shifts at the QA/1T-MoS_2 interface. However, our experimental evidence shows that this phase transition **progresses at a relatively slow rate** under ambient conditions, with 1T-MoS_2 remaining dominant for up to 75 days (**Supplementary Figure 3**). Furthermore, to ensure the reliability of our results, we **exclusively used MoS_2 nanosheets and membranes prepared within 60 days**, a timeframe during which 1T-MoS_2 remains the predominant phase. This experimental control supports the robustness of our interpretations, particularly those based on XPS analyses. These findings reaffirm the validity of the electron density shifts observed at the QA/1T-MoS_2 interface and the stability mechanisms proposed in our study.

Reviewer #2 (Comments for the Author)

General comments: *The manuscript has been reviewed previously. All my concerns have been well-solved and the paper has revised a lot. Thus, it has been in a good shape, which can be published now.*

Response: We appreciate the reviewer's recognition of our work.

Reviewer #1 (Remarks to the Author)

General comments: The responses and revisions are fine.

Response: We appreciate the reviewer's careful evaluation of our manuscript and revisions, and valuable input throughout this process.
